# Host Species Mediate Distinct Seed Microbiome Responses to Restoration

**DOI:** 10.1007/s00248-026-02802-6

**Published:** 2026-06-13

**Authors:** Allison A. Mertin, Linda L. Blackall, Douglas R. Brumley, Edward C. Y. Liew, Marlien van der Merwe

**Affiliations:** 1 Research Centre for Ecosystem Resilience, Botanic Gardens of Sydney, Sydney, Australia; 2 School of BioSciences, The University of Melbourne, Parkville, VIC 3010 Australia; 3 School of Mathematics and Statistics, The University of Melbourne, Parkville, VIC 3010 Australia

**Keywords:** Seed microbiome, Restoration, Microbial diversity, Plant growth promotion, Seeds

## Abstract

Microbial diversity is a key driver of ecosystem function, yet it remains poorly integrated into ecological restoration frameworks. While seeds are the primary dispersal unit of plants and the foundation of most restoration programs, the microbial communities they carry are rarely considered. Here, we assess whether sites that have undergone restoration have altered seed-associated microbial communities. We do this by comparing bacterial and fungal seed microbiomes across natural and restored landscapes. Using a landscape-scale sampling design, seeds were collected from multiple plant host species and populations across 41 sites spanning a broad latitudinal gradient. High-throughput sequencing of the 16S rRNA gene and ITS2 region was used to characterise bacterial and fungal communities, respectively, and differences in diversity, composition, and network structure were assessed using multivariate and network-based approaches. Seed microbiomes differed between natural and restored sites, but the magnitude and nature of these changes varied among host species. Restored sites were associated with shifts in microbial diversity, community composition, and network structure, including changes in the retention of putative keystone taxa. In some species, restoration was linked to pronounced restructuring of seed-associated microbial communities, whereas in others, microbiomes remained comparatively stable. Together, these results demonstrate that restoration can alter seed microbial communities in ways that are not consistently predicted by soil-focused restoration outcomes and that host identity mediates these responses. Incorporating seed microbiome data into restoration monitoring may therefore provide a complementary and previously overlooked indicator of restoration success, with implications for improving plant germination and health.

**Supplementary Information:**

The online version contains supplementary material available at 10.1007/s00248-026-02802-6.

## Introduction

Microbial diversity is an important attribute of functioning and productive ecosystems [1, 2]. Changes in microbial diversity could alter ecosystem processes such as litter decomposition, organic matter mineralisation, nitrogen uptake and primary production [3]. Altering these key processes may result in degradation and loss of these ecosystem functions. Therefore, it is important to ensure that microbial diversity is captured as a key terrestrial ecosystem attribute if degraded sites are to be restored to functional, resilient ecosystems. However, microbial diversity is often overlooked when restoring ecosystems [4] and is infrequently included in post-rehabilitation or restoration monitoring programs. Yet, ecological restoration aims to create self-sustaining, diverse and resilient ecosystems [5, 6] and ecosystem recovery is only seen as complete once all key attributes mirror that of a healthy natural reference ecosystem [7].

Reference ecosystems serve as models for identifying the target state of restoration projects, providing a basis for planning, monitoring, and assessing outcomes [8]. They are typically developed using information from existing reference sites, historical records, and predictive data to describe the composition, structure, and function of the ecosystem prior to degradation [8]. Studies have adapted this approach to monitor microbial diversity changes in soil by characterising the microbial community structure and diversity in both the degraded and reference sites [9]. This allows for the identification of specific microbial taxa or functional groups that can serve as indicators of ecosystem health and restoration progress [10, 11]. By incorporating microbial data into restoration monitoring, practitioners can gain a more comprehensive understanding of ecosystem recovery, including microbial processes that are crucial for overall ecosystem function and resilience [12].

Efforts to integrate microbial diversity into terrestrial restoration have focused on the below-ground microbial component of ecosystems [13–16], with above-ground microbial components rarely considered. Seed microbes, those existing within living seed tissue, without causing detriment to the host, comprise an important component of the plant microbiome but are the least studied [17]. This component of the microbiome can form the primary inoculum for the initial plant microbiome, having the capability to colonise the emerging seedlings with potential impacts on plant fitness [18]. Seed microbes provide benefits to the host plant especially during early lifecycle stages [19] and in the spermosphere (the soil and microbes around a dormant or germinating seed) [20]. These benefits include breaking seed dormancy [21], reducing seed predation [22], promoting germination [23–25] and improving seedling establishment [26]. Seed microbiomes also play a key functional role in mediating biotic and abiotic stress during the early stages of plant development. Seed-associated microbes can suppress pathogens through competitive exclusion, antimicrobial compound production, and priming of host immune responses, thereby reducing disease incidence during germination and early growth [27, 28]. However, seed microbes have also been implicated as latent pathogens and latent saprotrophs [29]. In addition, many seed microbes contribute to abiotic stress tolerance by enhancing nutrient acquisition, producing phytohormones such as indole-3-acetic acid, and modulating plant stress signalling pathways, which can improve seedling vigour under drought, salinity, or nutrient-limited conditions [30–32].

Seed microbial diversity remains relatively unexplored despite seeds being the major dispersal agent of plants, and the major source of material used in restoration practices [33].When seeds disperse across the landscape, the associated seed microbial community travel with them, however the microbiome contained within the seeds can be altered through processes associated with seed harvesting, storage and distribution [34]. However, little is known about whether the microbiome is altered through restoration practices [33]. Due to the importance that seed microbes play in host germination and establishment and the vital role of seed in restoration, identifying what seed microbial species are present in natural ecosystems and whether they are retained or altered during restoration practices is crucial. Such information may help us understand which components are missing and if mitigation practices should be considered.

Recovery trajectories of microbial communities in restored ecosystems are typically slow and lag behind vegetation, with soils often requiring years to decades to converge toward reference states due to dispersal limitation, legacy effects, and environmental filtering [35–38]. Recent work suggests that early microbiome assembly is highly dynamic, with seed-associated microbes acting as an initial inoculum but interacting with soil-derived communities during seedling establishment [39–41]. In restoration settings, where soils are often depauperate or functionally altered [42], reliance on soil-derived communities may not ensure beneficial outcomes, potentially increasing the importance of seed-associated microbiota.

Without baseline knowledge on the structure and diversity of microbial communities in healthy natural reference ecosystems, successfully incorporating microbial diversity into restoration practices is challenging [12]. The structure of a microbiome comes from complex interactions and inter-relationships between members and has substantial effects on its functioning [43]. Microbial co-occurrence network analysis [44] has been used to understand how microbial community structure can change in response to restoration previously within soil [45–47] and root microbiomes [48]. Within seed microbiome studies, network analyses have shown that domestication alters the rice seed microbiome structure [49] and the microbiome of multiple cereal crops [50], and it has been used to identify putative keystone species [51] and fungal bacterial inter-kingdom interactions [52]. The effect of restoration on the structure of seed microbiomes using this approach has not yet been explored.

Investigations of seed microbial communities of natural ecosystems are rarely at a broad spatial scale, and mostly focus on a single host species [27, 52]. Understanding how microbial communities differ amongst populations of a host species and between different host species is important as seed origin is a key consideration in restoration [53]. Landscape-scale experimental designs that survey multiple populations of a host, and multiple host species, allows for robust identification of core microbial taxa. These are microbes that are thought to play an important role in the functioning of the community, and within seed microbiomes it is proposed core microbes are vertically transmitted to the next generation [54]. Thus, determining the seed microbiome across space will provide not only knowledge of how communities change with geographic distance, but also allow for identification of core seed taxa, shared amongst populations or host species.

In this study we employed a landscape-scale comparative approach to investigate whether the bacterial and fungal seed microbiome was similar between restored and natural sites. We hypothesised that previously degraded, and then restored sites would have an altered microbial community relative to natural reference sites, due to a potential lowered diversity in the seed used to revegetate the site [34], but additionally due to potential differences in the soil microbiome between degraded and natural sites [55, 56] and reduced plant species diversity at restored sites possibly impacting the pathways by which seed microbes can be acquired [57]. This knowledge has implications for identifying whether seed microbiomes may complement soil microbiota surveys in restoration monitoring and assessment, and for seed collection and storage in seed banks.

## Methods

### Study System and Sampling Design

**Fig. 1:**
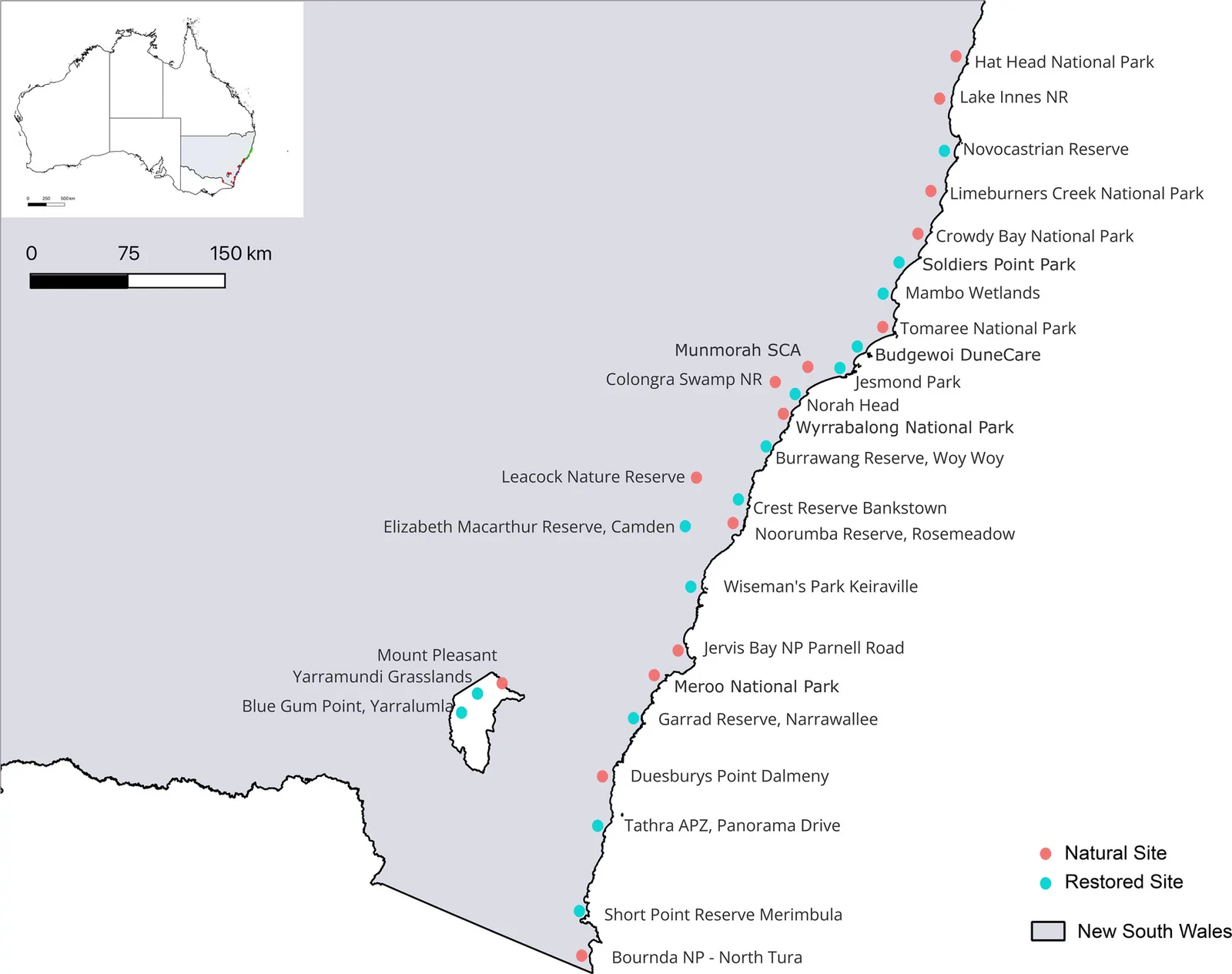
Map showing location of natural (pink circles) and restored sites (light blue circles) where seed was collected from the three hosts species: *Melaleuca quinquenervia*, *Microlaena stipoides* and *Themeda triandra* within New South Wales (grey region) and the Australian Capital Territory (white inset region), Australia during 2020/2021

Seed was collected from three plant species often used in restoration, two grasses (*Microlaena stipoides* and *Themeda triandra*) and a tree species (*Melaleuca quinquenervia*). These plant species are wide spread and locally abundant [58]. Seeds were collected during the summer (December to February) of 2020/2021 from a total of 41 sites including 21 ‘natural’ (undisturbed and in protected areas) and 20 ‘restored’ (previously degraded and disturbed and at some point, revegetated) sites, within New South Wales, Australia. Information on restoration history, including restoration age, land-use history, and the specific drivers of degradation, was not consistently available across all sites due to limited records from land managers and landowners. As a result, restored sites in this study represent a heterogeneous set of conditions, encompassing a range of restoration ages and variable prior land-use histories. Where available, site-specific metadata have been provided in the Supplementary Materials (Table S1). To reduce the influence of environmental variation, we used a paired sampling design in which restored and natural sites were selected in close geographic proximity and within the same plant community type (Fig. 1; Table S1). This approach was intended to minimise differences in broad-scale abiotic and biotic conditions (e.g., climate, soil type, and vegetation composition) between paired sites, thereby strengthening inferences about associations between restoration and seed microbiomes. However, we did not directly measure fine-scale environmental variables (e.g., soil chemistry, moisture, disturbance history, or land-use legacy), and therefore cannot fully exclude the possibility that unmeasured environmental differences contributed to the observed patterns. As such, our results should be interpreted as associations between restoration status and seed microbiome variation, rather than causal effects.

Three mature spikelets from each of eight visually disease free plants at least 5 m apart were collected for the grasses at each site. For the tree, *M. quinquinervia*, woody capsules were collected using extended pole pruners from each of eight trees separated by more than 10 m at each site. Seeds were removed from the capsules and spikelets and transferred to −80 °C for storage until processing.

### DNA Extraction and Illumina MiSeq™ Sequencing

Grass seeds were de-husked, whilst woody capsules and chaff were removed from *M. quinquenervia* seeds. Seeds were pooled by maternal plant and serially immersed under agitation in a bench top tube rotator (VWR, #10,136–084) in 0.5% household bleach for 2 min followed by rinsing with sterile de-ionised water twice, followed by immersion in 70% ethanol for 2 min, followed by rising in sterile de-ionised water twice. Seeds were then lyophilised for four hours and 50 mg from each sample was ground under liquid nitrogen in a mortar and pestle. DNA was extracted using the Macherey Nagel™ Nucleomag Plant Extraction Kit as per the manufacturer’s instructions. Three extraction controls that contained no sample material were included.

A two-round amplification process was used to amplify the DNA samples, and peptide nucleic acid (PNA) clamps added to supress host DNA amplification following [59]. For the first-round PCR, extracted DNA and extraction controls were amplified in triplicate using bacterial primers with Illumina Nextera adapters (underlined) designed at the Walter and Eliza Hall Institute (WEHI) targeting ~ 291 bp of the V4 region of the 16S rRNA gene: 515f 5’-GTGACCTATGAACTCAGGAGTCGTGYCAGCMGCCGCGGTAA-3’ [60]; 806r 5’-CTGAGACTTGCACATCGCAGCGGACTACNVGGGTWTCTAAT-3’ [61]. Additionally, fungal primers with Illumina Nextera adapters (underlined) designed at the Walter and Eliza Hall Institute (WEHI) targeting ~ 330 bp of the fungal nuclear ribosomal internal transcribed spacer region 2 (ITS2): fITS7 5’-GTGACCTATGAACTCAGGAGTCGTGARTCATCGAATCTTTG-3’ [62]; ITS4 5’-CTGAGACTTGCACATCGCAGCTCCTCCGCTTATTGATATGC-3’ [62] were used. Each first-round PCR reaction contained 12.5 μL of MyTaq HS polymerase Mix (Bioline, Meridian Bioscience, France), 2 μL of DNA template (2–5 ng/μL), 0.4 μM of each primer, 1 μM each of pPNA 5’-GGCTCAACCCTGGACAG-3’ [63] and mPNA 5’-GGCAAGTGTTCTTCGGA-3’ [63, 64] clamps and molecular grade nuclease free water to 25 μL. PCR conditions were: 1 cycle × 95 °C for 3 min; 18 cycles × 95 °C for 15 s, 75 °C for 10 s (for PNA clamp annealing), 55 °C for 30 s, and 72 °C for 30 s; 1 cycle × 72 °C for 7 min. PCR negative controls (n = 3) were also amplified whereby the sample template was replaced with molecular grade water to identify contaminants introduced during the PCR. The triplicate PCR products from each sample and PCR negative controls were then pooled.

Remaining library preparation steps were performed as per Mertin, Blackall, Brumley, Liew and van der Merwe [59]. Briefly, 20 μL of each PCR product pool was used as a template for the second-round PCR. The products were purified by size selection using Ampure XP magnetic beads (Agencourt, Becker Coulter, Australia), as per the manufacturer’s instructions, with a final clean with 70% ethanol. The purified second-round PCR product was resuspended in 40 μL molecular grade nuclease free water. Ten μL of this solution was used in the second-round PCR, combined with 10 μL of MyTaq HS polymerase Mix (Bioline, Meridian Bioscience, France), and 0.25 μM of each forward and reverse custom barcodes designed at the WEHI. PCR conditions were: 1 cycle × 95 °C for 3 min; 24 cycles × 95 °C for 15 s, 60 °C for 30 s, and 72 °C for 30 s; 1 cycle × 72 °C for 7 min.

Amplification was assessed at the first and second-round PCR stages through separation of the PCR products via electrophoresis on a 1% agarose gel, visualised under ultraviolet light after staining with SYBR Safe DNA gel stain (Thermo Fisher Scientific, MA, USA). Sequencing libraries were created by pooling 5 μL from each reaction and performing a final bead clean up as performed previously. Each library was checked for quality and quantity to guide pool normalisation (2200 TapeStation, Agilent Technologies, U.S.A.), then sequenced on a single Illumina MiSeq run using v3 (2 × 300 bp) chemistry at the WEHI, Melbourne, Australia.

Due to the large number of samples sequenced for two loci, multiple Illumina sequencing runs had to be performed. To assess potential batch effects, mock communities, (comprised of DNA from pure cultured isolates of known identity and concentration) were used and sequenced in multiple runs to determine batch effects. Two-way analysis of variance (ANOVA) tests implemented in the software JMP v.17.2.0 were used to assess whether all species were recovered in each sequencing run, whether there was significant variation in the number of reads assigned to each mock community amplicon sequence variant (ASV) between the three sequencing runs, and separately whether the read number varied between technical replicates. The results from these analyses are presented in Supplementary Results.

### Quality Control and Read Processing

De-multiplexed paired-end sequences were processed using the Quantitative Insights Into Microbial Ecology QIIME2 pipeline version 2021.8 [65]. Cutadapt [66] was used to remove primers, using an error rate of 0.20. DADA2 [67] was used to merge trimmed forward and reverse reads, denoise and remove poor-quality sequences (mean Qscore < 30), perform dereplication and eliminate chimeras. Sequences were assigned to amplicon sequence variants (ASVs) (sequences sharing 100% sequence similarity). Taxonomy was assigned using the feature-classifier plugin in QIIME2. Taxonomy for each 16S rRNA ASV was assigned against the SILVA SSU database (version 138) [68] trained with a naïve Bayes classifier against the same V4 16S region targeted for sequencing for bacteria and against the UNITE v8 database [69] using the ITS2 region for fungi.

All data were analysed in R v4.2.3 [70], unless otherwise stated, and statistical significance was determined at α = 0.05. All analysis R code, QIIME2 code and CoNET session and parameter files are available at https://github.com/amertin-sudo/Seed-Microbiomes-and-Host-Species and archived on Zenodo (10.5281/zenodo.20281527). Analyses were performed separately for bacteria and fungi, excepting in the network analyses where datasets were combined to allow for identification of inter-kingdom interactions. Potential contaminant ASVs were identified and removed from the dataset according to their abundance in the extraction (n = 3) and PCR (n = 3) negative controls relative to the seed samples using the prevalence method in the R package ‘decontam’ [71] with *p* = 0.1. Rarefaction curves were visualised using the *rarecurve* function in ‘vegan’ [72]. Results for these analyses are presented in Supplementary Results.

### Diversity and Community Composition Comparisons

Three diversity metrics: richness, Shannon’s diversity index and Simpson’s 1-D index, were calculated for each host species for each of the site types (restored and natural). To allow for even sampling amongst treatment groups, based on constructed rarefaction curves, samples were rarefied to 8,000 reads per sample for the bacterial community. Rarefaction of bacterial datasets resulted in loss of over half of the samples; therefore, non-rarefied data were used for the bacterial diversity metric comparisons. For the fungal community, reads were rarefied to 15,000 reads per sample, resulting in two samples removed from the analysis. Rarefaction was used only for alpha diversity analyses to standardise sampling effort across samples and to reduce bias arising from uneven sequencing depth. While rarefaction has recognised limitations, including the loss of data [73], it remains a widely used approach for estimating diversity when sequencing depth varies among samples [74, 75]. For comparison, these analyses were also run on the complete (non-rarefied datasets) (Table S5b). The distribution of the diversity metric data was assessed for normality through inspection of the frequency distribution of the residuals and inspection of quantile plots. A normal curve was fitted to the frequency distribution plot and additionally a Shapiro-Wilks test was undertaken to test whether the residuals are normally distributed. As the data conformed mostly to a normal distribution, differences in these diversity metrics between host species and the site types and their interaction were tested using separate two-way ANOVAs implemented in the packages ‘nlme’ [76] and ‘emmeans’ [77] using the functions *lm* and *pairs* for post hoc pairwise Tukey’s HSD comparisons between significant factors.

Abundance matrices (comprising data from the non-rarefied datasets) were log transformed using the *decostand* function in ‘vegan’ [72] to give rare and abundant taxa more even representation in the analysis. Bray Curtis dissimilarities were calculated from the transformed abundance matrices, as this dissimilarity measure has been reported as well suited to assess microbial communities [78, 79]. Bray Curtis dissimilarities between samples were visualised using principal coordinate analyses (PCoA) to assess clustering of samples based on site type, for each host species. To assess differences in microbial community composition between restored and natural sites, and whether these effects varied among host species, we performed a permutational multivariate analysis of variance (PerMANOVA) using the *adonis2* function in the package ‘vegan’ [72]. The model included host species, site type (restored or natural) and their interaction. Given the paired sampling design (natural and restored sites sampled as matching pairs), permutations were constrained within site pairs, therefore accounting for non-independence among paired samples. Analyses were conducted using 999 permutations. Homogeneity of multivariate dispersion was assessed using the *betadisp* function prior to PERMANOVA, testing for each of the main effects in the model and their interaction. Where appropriate, post hoc pairwise PERMANOVA comparisons were conducted using the *pairwise.adonis2* function from the ‘pairwiseAdonis’ [80] package. P values were adjusted for multiple comparisons using the default Bonferroni correction [81].

A similarity percentages (SIMPER) [82] analysis in the software Primer-e v7 was performed on log transformed abundance matrices to assess whether any of the host species showed greater community dissimilarity between the two site types, and to identify the bacterial and fungal ASVs that contributed most to the dissimilarity between the site types.

### Identification of Core ASVs

To identify whether core ASVs present in natural sites were detected as core ASVs in restored sites, core microbial ASVs were identified across each site type. The approach of Shade and Handelsman [83] was used to identify core ASVs on consideration of their occupancy (i.e. detection across samples) and abundance amongst samples of each host species. ASVs were defined as “core” if they were in greater than 50% of samples and had a mean relative abundance greater than 0.01 (on average the taxon represents equal to or greater than 1% of the total community in each sample). The application of a moderate (greater than 50% occupancy) cutoff threshold follows previous comparative and conceptual treatments of core definitions [84, 85], and the abundance cutoff was applied to exclude very low abundance taxa. Core species were identified for each site based on the above thresholds, separately for each host species, using custom scripts in R v4.2.3 [70], and visualised using the package ‘pheatmap’ [86].

### Identification and Functional Guild Assignment of Indicator ASVs

To determine whether there were ASVs that had a significant association with either natural or restored sites, an indicator species analysis was performed using the *multipatt* function in the package ‘indicspecies’ [87]. The analysis was performed separately for each host at minimum positive predictive value (At) = 0.1, a minimum sensitivity (Bt) = 0.1, and alpha = 0.05 for bacteria and fungi separately. Further analyses were only conducted on indicator species with resulting IndVal indicator values > 0.30, and significance values of *p* < 0.05.

To identify what ecological role each indicator species may play within the seed microbiome at natural and restored sites we undertook assignment of guilds to the ASVs identified as indicator species in the previous analysis. Bacterial guilds were manually assigned to each indicator ASV using the plant-associated bacteria database v.1.02 (PLaBA-db) [88]. In PLaBA-db bacteria are assigned to one of six plant spheres (i.e. carposphere, phyllosphere), and to one of four ‘lifestyles’ (plant/lichen symbiont, plant growth promoting bacteria, plant pathogen or human/animal pathogen). Fungal guilds were assigned to indicator ASVs based on the primary lifestyle assigned within the fungal functional guild database FungalTraits [89] implemented using the *trans_func* function in the ‘microeco’ package [90]. In FungalTraits fungal genera are assigned to one of 30 primary lifestyles based on trophic strategies such as pathogen, saprotroph, symbiotroph or mycoparasite [89].

### Co-occurrence Network Analysis

Co-occurrence patterns in the combined fungal and bacterial datasets were assessed by performing network analysis on log transformed abundance data in the software CoNet v. 2.6.1 [91] using Pearson’s correlation and Bray–Curtis dissimilarity measures. These measures allowed for assigning a positive (co-occurrence) or negative (mutual exclusion) sign to a predicted relationship, which reflects whether the abundance distribution of the two ASVs are significantly more similar or dissimilar than expected at random. Bacterial and fungal ASVs that had less than 100 reads were removed from the network analysis, representing less than 0.01% of the dataset. Separate networks were constructed for each host species from each site type (natural or restored).

For each edge and association measure, permutation and bootstrap distributions were generated with 100 iterations. Network integration was performed using the intersection merge strategy, retaining only edges supported by both measures. Statistical significance of associations was assessed by bootstrap resampling with 100 iterations, and *p*-values for correlation-based measures were calculated using the Fisher’s Z transformation. Multiple testing correction was applied using the Bonferroni method, and only associations with corrected edge significance values below 0.05 were retained. The nodes in the constructed networks represent ASVs, and edges between the nodes represent strong and significant correlation in co-occurrence or mutual exclusion between ASVs. Networks were visualised in Cytoscape v3.10.2 (https://cytoscape.org/index.html) and network layout was implemented using the yFiles (yworks. https://www.yworks.com/products/yfiles) radial layout algorithm in Cytoscape v3.10.2.

To verify the robustness of the constructed networks, the networks were compared against their randomised version (baseline null models) [92]. Random networks were constructed using the Babarasi-Albert model, using the Network Randomizer v1.1.3 plugin [93] in Cytoscape v3.10.2. Average differences between the empirical and randomised network for all the topological parameters were calculated using the Network Randomizer v1.1.3 plugin. Average differences of greater than zero indicate that the empirical networks differ from the random ones [93].

To investigate changes in community structure the NetworkAnalyzer plugin [94] in CytoScape v3.10.2 was used to calculate seven network topology parameters for each node, focussed on assessing changes in network connectivity and overall network structure. Edge statistics were calculated so that significant inter-kingdom and infra-kingdom interactions could be investigated. Descriptions of the parameters used is listed in Table S2. Network connectivity was assessed as a combination of four topological parameters, namely: number of degrees, clustering co efficient, closeness centrality and the betweenness centrality, following Lee, Kim and Lee [49] and Banerjee, Walder, Büchi, Meyer, Held, Gattinger, Keller, Charles and van der Heijden [48]. Separate pairwise t-tests were used to compare average node topological parameters between natural and restored networks for each host species in the software JMP v.17.2.0.

Cluster detection analysis was used to identify densely connected regions within each network, allowing for identification of highly interconnected groups of ASVs [95]. The Molecular Complex Detection (MCODE) plugin [96] in Cytoscape v3.10.2 was used. For cluster detection in each network, degree cutoff was set to two and cluster finding was performed using the ‘Haircut’ mode, with a node cutoff score of 0.2, a K-Core value of 2 and a maximum depth of 100. The clusters were formed and ranked based on the MCODE cluster score [96].

Keystone taxa are species that have a disproportionate role in the community relative to their abundance and putative keystone taxa can be detected in microbial communities through calculation of network topological parameters such as degree, closeness centrality and betweenness centrality [97]. ASVs in each network with degrees higher than 4, closeness centrality higher than 0.28, and betweenness centrality lower than 0.18 were selected as the keystone taxa, following the approach of Banerjee, Walder, Büchi, Meyer, Held, Gattinger, Keller, Charles and van der Heijden [48]. A single set of cut-off values were used for consistent comparison between species × site type networks.

## Results

### Read Coverage

Illumina MiSeq sequencing of the V4 region of the 16S rRNA gene, and ITS2 region produced 15,280,839 and 16,397,092 reads, respectively across seed samples from *Melaleuca quinquenervia* (n = 97), *Microlaena* *stipoides* (n = 121) and *Themeda triandra* (n = 119), extraction blanks (n = 9, 7), PCR blanks (n = 12, 9) and mock samples (n = 9), for a total 383 samples. After de-noising, merging and chimera detection, and removal of reads assigned as chloroplast or mitochondrial, a total of 4,599,646 bacterial and 12,522,578 fungal reads were retained. After quality control, bacterial sample sizes were 84, 120, and 118 for *M. quinquenervia*, *M. stipoides*, and *T. triandra*, respectively; fungal sample sizes were 97, 121, and 119. Rarefaction curves for the datasets, after the removal of contaminant sequences approached the asymptote for all bacterial and fungal samples, at 8,000 and 15,000 reads per sample, respectively (Fig. S1).

Mock community bacterial and fungal controls showed that for each sequencing run all taxa that were present in the mock community were retrieved. The highest variation between technical replicates for the mock bacterial samples was seen in run three where technical replicates saw a variation of 3,216 reads of *Agrobacterium larrymoorei* (Supplementary Results)*.* For the fungal community highest variation between technical replicates was seen in run two with a variation of 3,372 reads between replicates of *Schizophyllum commune* (Supplementary Results).

### Community Composition and Comparisons Between Site Types

A total of twenty-seven bacterial and four fungal phyla were identified from the three hosts. Proteobacteria was the dominant bacterial phylum comprising 90.34% of bacterial reads, Actinobacteria (3.21%), Firmicutes (3.16%) and Bacteriodota (1.68%), were the next most abundant, with the remaining 23 phyla each comprising less than 1% of the bacterial dataset. *Erwiniaceae* was the dominant bacterial family (45.55% of all reads), with *Pseudomonaceae* (16.34%), *Enterobacteriaceae* (8.42%), *Xanthomonadaceae* (3.27%) and *Rhizobiaceae* (3.22%) the next most abundant. Ascomycota was the dominant fungal phylum comprising 92.51% of fungal reads, Basidiomycota comprised 7.48%, whilst Mucoromycota and Chytridomycota each comprised less than 0.1% of the fungal dataset. *Pleosporaceae* was the dominant fungal family (29.81% of all reads), with *Didymellaceae* (12.93%), *Cladosporiaceae* (8.88%), *Botryospheriaceae* (4.55%) and *Cystostereaceae* (4.17%) the next most abundant.

**Fig. 2:**
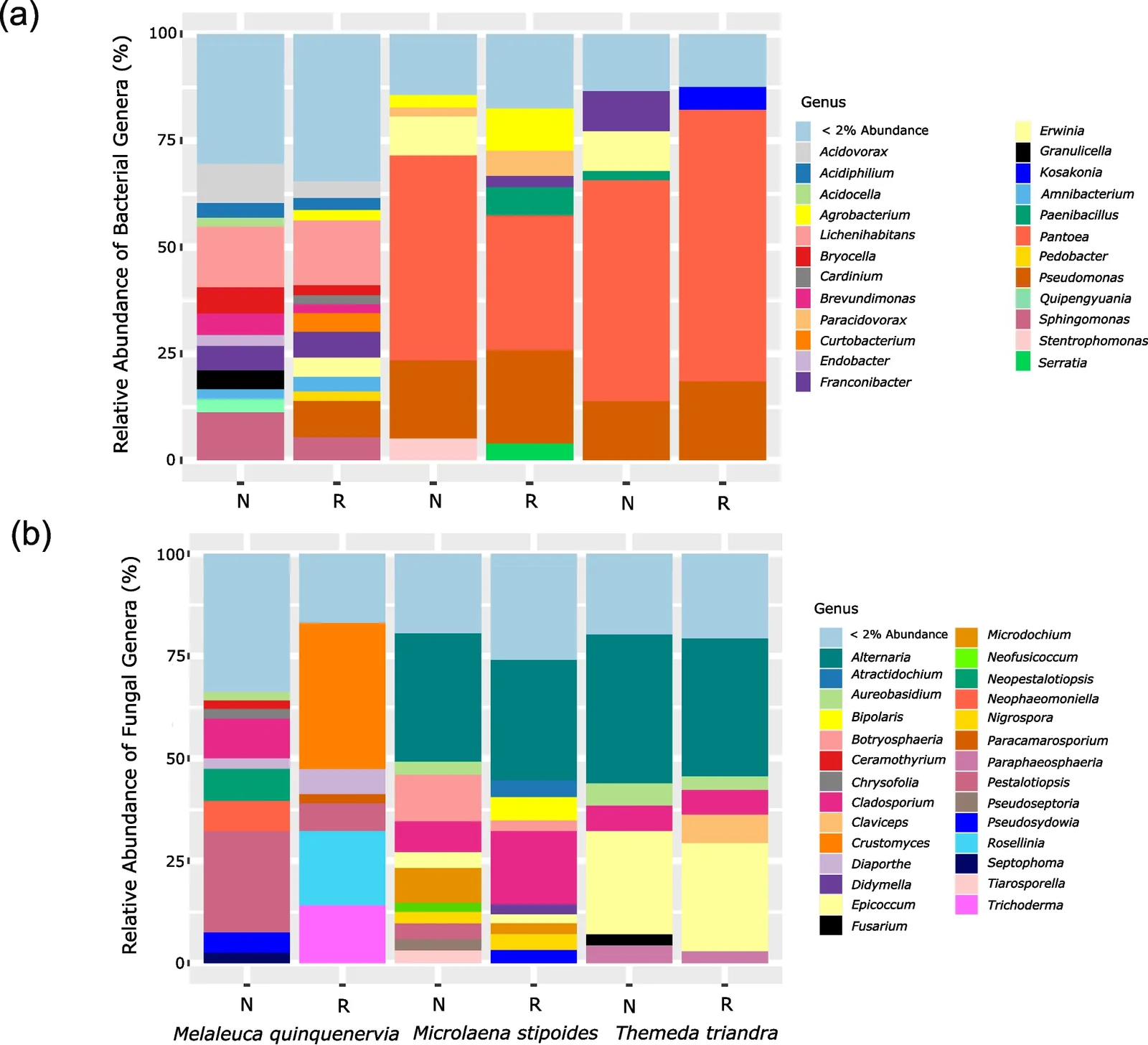
Relative abundance (%) of 16S rRNA gene and ITS2 region sequences assigned to (**a**) each bacterial and (**b**) each fungal genus identified from seed collected from natural (N) and restored (R) sites of *Melaleuca quinquenervia*, *Microlaena stipoides* and *Themeda triandra*

The bacterial and fungal community taxonomic composition differed between natural and restored sites of all the three host species (Bacteria: PerMANOVA_SiteType_ *F* = 5.15, d.f. = 1,318, *p* = 0.001, permutations = 999; Fungi: PerMANOVA_SiteType_ *F* = 6.54, d.f. = 1,331, *p* = 0.001, permutations = 999) (Fig. 2, Table S3). Natural and restored sites of *M. quinquenervia* both had *Lichenihabitans* comprising the most abundant bacterial genus (comprising 15.3%, and 14.2%, respectively). However, the dominant fungal genus for *M. quinquenervia* natural sites was *Pestalotiopsis* (24.8% relative abundance) and for restored sites *Crustomyces* (35.8% relative abundance) (Fig. 2). *Microlaena stipoides* and *T. triandra* had species of *Pantoea* and *Pseudomonas* as the abundant bacterial genera in both site types. *Pantoea* comprises 48% and 52% of sequences from natural, and 32% and 64% of sequences from restored sites of *M. stipoides* and *T. triandra* respectively. Despite being the next most abundant genus identified in both the grasses, *Pseudomonas* comprises only 18% and 14% of natural and 22% and 19% of restored sites for *M. stipoides* and *T. triandra* respectively (Fig. 2)*.* Both grasses have *Alternaria* as the most abundant fungal genus in each site type (*M. stipoides* (31% natural, 29.15% restored), *T. triandra* (36.5% natural, 33.7% restored)) (Fig. 2).

**Fig. 3:**
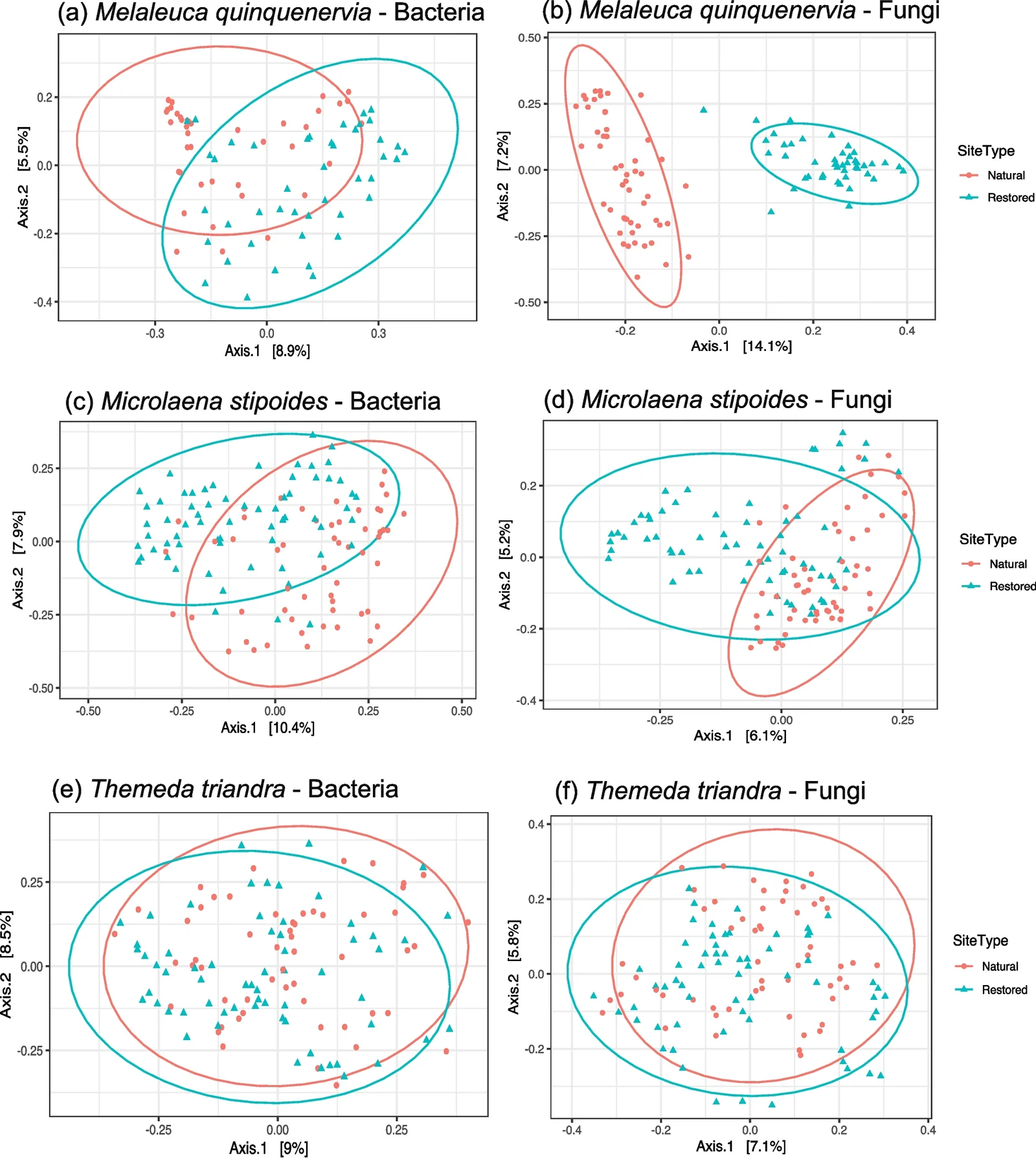
Principal coordinates analysis (PCoA) showing Bray Curtis dissimilarities between seed bacterial (a, c & e) and fungal (b, d & f) communities collected from natural (pink circle) and restored (light blue triangle) sites of three host plant species: *Melaleuca quinquenervia* (a, b), *Microlaena stipoides* (c, d) and *Themeda triandra* (e, f). Ellipses represent 95% confidence intervals based on 999 permutations of the data

Samples clustered based on site type for *M. quinquenervia* and *M. stipoides* in the PCoA, in particular the fungal communities in *M. quinquenervia* showed little overlap, whereas for *T. triandra* clustering was less defined by site type (Fig. 3). PC axis 1 explained between 8.9% to 10.4% of the bacterial community variation, whilst for the fungal communities the PC1 axis explained between 6.1 to 14.1%. The results of the PCoA were supported by the SIMPER analysis which showed that *M. quinquenervia* had the largest dissimilarity (96.60% bacterial dissimilarity, 97.08% fungal) between natural and restored sites. *M. stipoides* had moderate dissimilarities of 91.62% bacterial and 88.34% fungal, whilst this was lower in *T. triandra* (87.28% bacterial and 84.05% fungal ). For the grasses, the differences in bacterial communities between the site types for each host was mostly explained by rare ASVs within each site type, whereas for *M. quinquenervia*, both abundant and rare ASVs contributed to differences between the site types (Table S4).

### Diversity Comparisons Between Site Types and Hosts

**Fig. 4:**
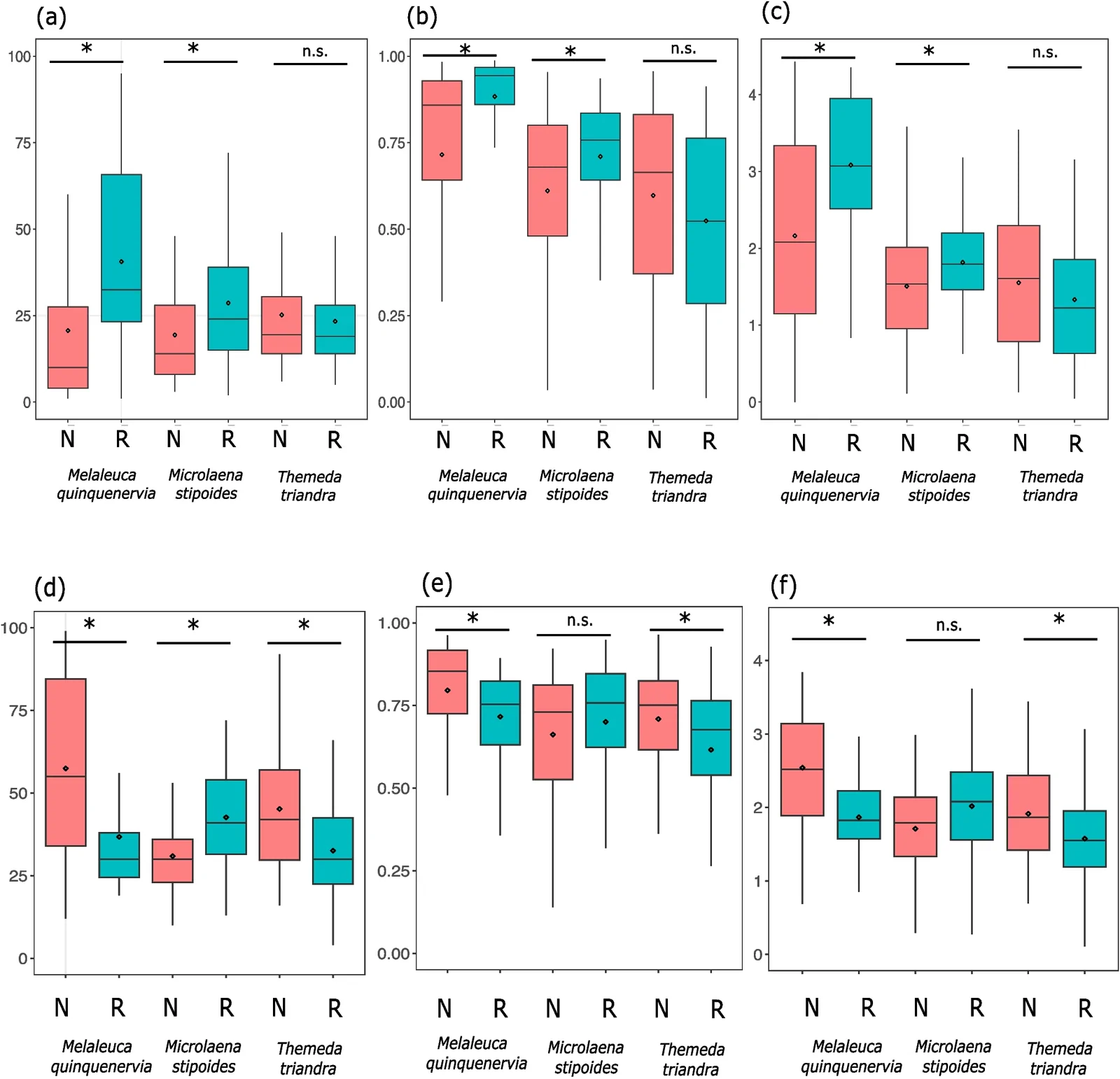
Box and whisker plots showing the mean (⋄), interquartile range and median (line) of the (a) observed bacterial richness (amplicon sequence variants), (b) Simpsons Diversity bacterial indices, (c) Shannon’s diversity bacterial indices (d) observed fungal richness, (e) Simpsons Diversity fungal indices, (f) Shannon’s diversity fungal indices for the three host plant species: *Melaleuca quinquenervia*, *Microlaena stipoides* and *Themeda triandra*, between natural (N) (pink) and restored (R) (light blue) sites. Significant differences between site types at *p* < 0.05 are indicated with an asterisk. n.s. represents comparisons where no significant difference was observed

A total of 3,148 bacterial and 6,721 fungal ASVs were identified from the three hosts combined. Restored sites had a higher bacterial richness and diversity compared to natural sites for *M. quinquenervia* (average of 40 and 20 ASVs per sample respectively) and *M. stipoides* (average 29 and 20 ASVs per sample respectively) (Fig. 4a-c; Table S5b), whereas for *T. triandra* both site types had similar levels of bacterial richness (average 19 and 20 ASVs respectively) and diversity (Fig. 4a-c; Table S5b).

For the fungal communities the opposite trend was shown where natural sites had a higher fungal richness and diversity for *M. quinquenervia* and *T. triandra*, whereas for *M. stipoides* the richness was higher in restored sites, whilst diversity levels were similar between the two site types (Fig. 4d-f; Table S5a).

*Melaleuca quinquenervia* housed the richest bacterial (average 30 bacterial ASVs per sample) and fungal community (average of 45 fungal ASVs per sample), and was the most diverse, whilst this diversity was lower and did not differ between the two grasses, excepting when using Simpsons diversity which showed higher bacterial diversity in *M. stipoides* (average = 0.66), indicating a more even spread of bacterial species in *M. stipoides* compared to *T. triandra* (Table S5a).

### Core ASVs Present in Native Seed

**Fig. 5:**
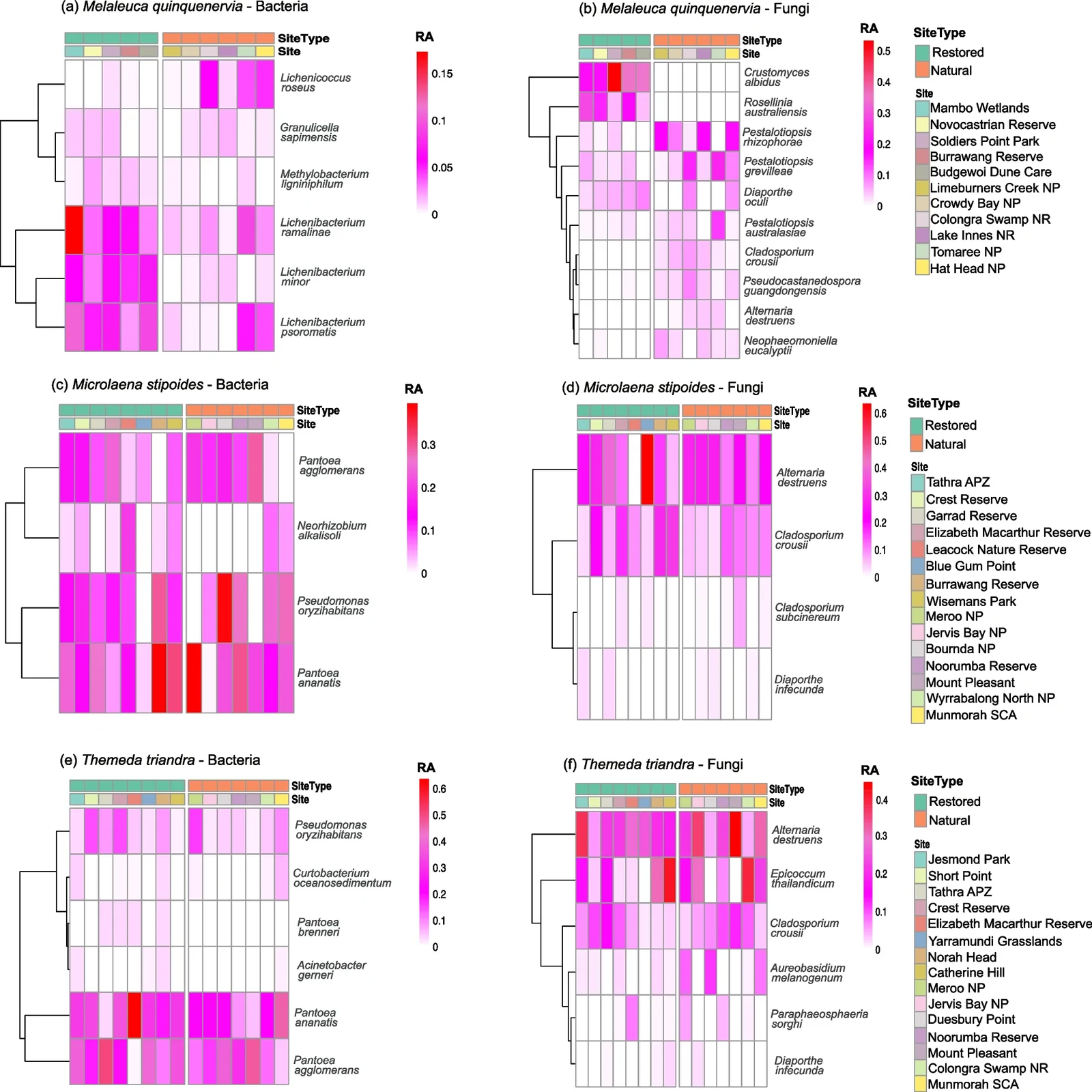
Heatmaps showing the relative abundance (RA) (pink to red) of core seed bacterial (a, c & e) and fungal (b, d & f) taxa at natural (orange squares) and restored (teal squares) site types of the three host species: *Melaleuca quinquenervia* (a & b), *Microlaena stipoides* (c & d) and *Themeda triandra* (e & f)

The number of core ASVs identified differed between each of the host species, and between the bacterial and fungal datasets. The host species also differed as to whether core ASVs from natural sites were detected in restored sites (Fig. 5). For *M. quinquenervia*, only two fungal ASVs (out of 16 core bacterial and fungal ASVs) (*Pestalotiopsis grevilleae* and *P. rhizophorae*) were identified as core ASVs in both site types, whilst the remaining core ASVs were only considered core of either site type (Fig. 5a-b).

*Microlaena stipoides* and *T. triandra* each had fewer core ASVs identified (8 and 12, respectively), but 50% (for *M. stipoides*) and 83% (for *T. triandra*) of the core ASVs identified in the natural sites were also detected as cores in the restored sites (Fig. 5c-f). *Pseudomonas oryzihabitans*, *Pantoea ananatis*, *Pantoea agglomerans*, *Alternaria destruens* and *Cladosporium crousii* were identified as core ASVs present in both grasses and amongst both site types (Fig. 5c-f).

### Indicator Species and Guild Assignment

**Fig. 6:**
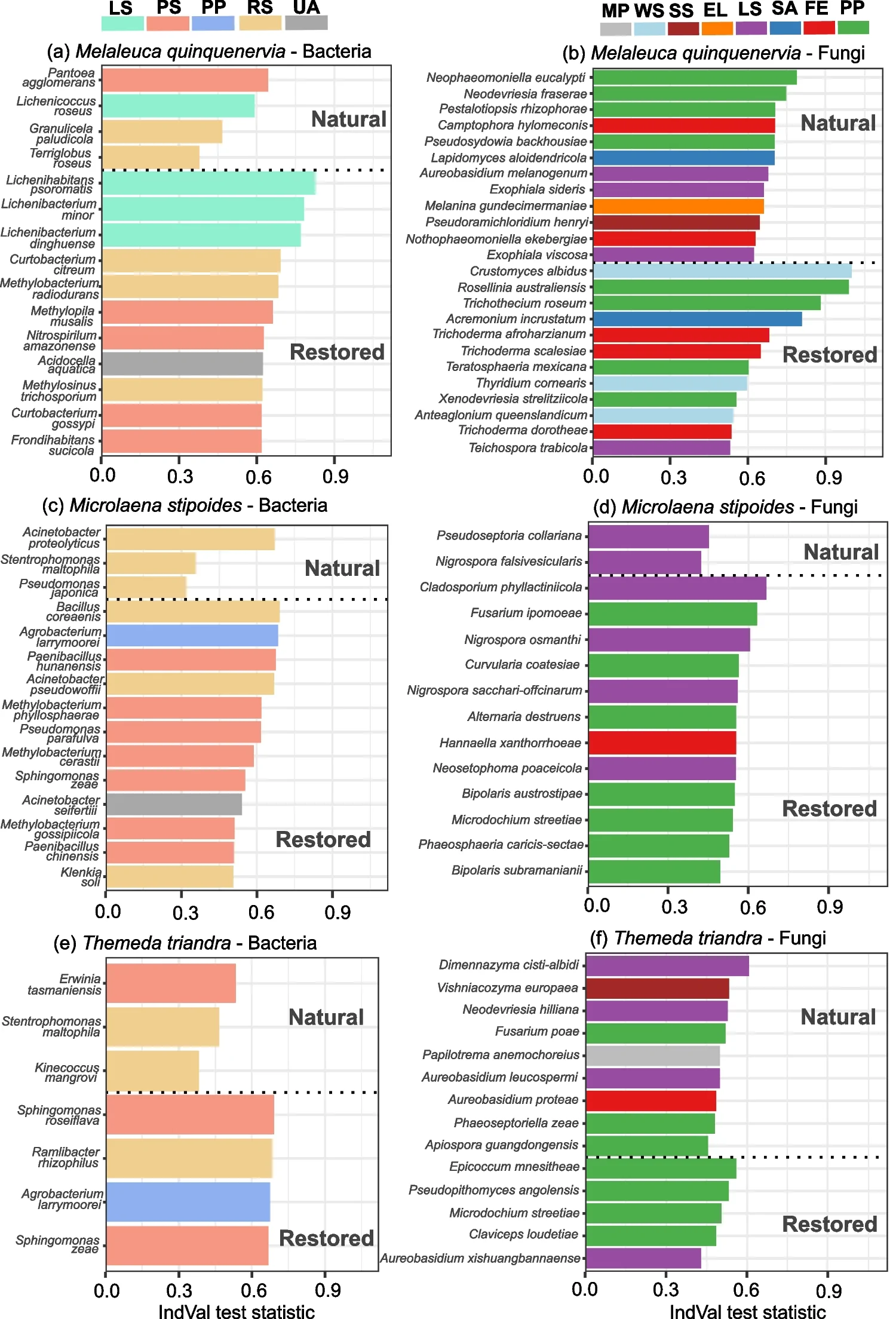
Top bacterial (a, c, e) and fungal (b, d, f) taxa identified as indicator species of natural or restored sites of the three host species. The strength of the significant (*p* < 0.05) association with that site type is shown by the IndVal test statistic where a larger IndVal is a stronger association. Colours show guild designation for that taxon using PLaBAse (bacteria) or FungalTraits (fungi) databases. LS = lichen symbiont, PS = plant symbiont, PP = plant pathogen, RS = rhizosphere, UA = unassigned. MP = mycoparasite, WS = wood saprotroph, SS = soil saprotroph, EL = endolichenic, LS = leaf litter saprotroph, SA = saprotroph, FE = Foliar endophyte

Four of the five plant bacterial guilds present in PLaBA-db were identified in the bacterial indicator species analysis, whilst eight of the 30 FungalTraits database fungal guilds were identified (Fig. 6, Table S6). The strongest associations identified overall were two fungal species *Rosellinia australiensis* (plant pathogen) and *Crustomyces albidus* (wood saprotroph) with restored sites of *M. quinquenervia* each having close to the maximum association value that can be assigned (IndVal 0.92, 0.94, respectively) (Fig. 6b). For the bacterial communities, species of *Lichenibacterium* and *Lichenihabitans* were assigned as lichen symbionts and methylotrophic species (*Methylopila, Methylosinus and Methylobacterium*) all of which were strongly associated (IndVal > 0.6) with restored sites of *M. quinquenervia* (Fig. 6a).

Rhizosphere-inhabiting bacteria and litter saprotrophic fungi were most strongly associated with natural sites of *M. stipoides,* whilst restored sites were strongly associated with multiple *Methylobacterium* species and other methylotrophic bacteria in addition to fungal plant pathogens and litter saprotrophs (Fig. 6c-d). Natural sites of *T. triandra* were strongly associated with *Erwinia tasmaniensis* (plant symbiont)*,* and a diverse range of fungal guilds whilst host plant symbionts (bacterial species of *Sphingomonas*) were associated with restored sites, as were predominantly fungal pathogens (Fig. 6e-f).

### Network Structure, Topology and Connectivity

**Fig. 7:**
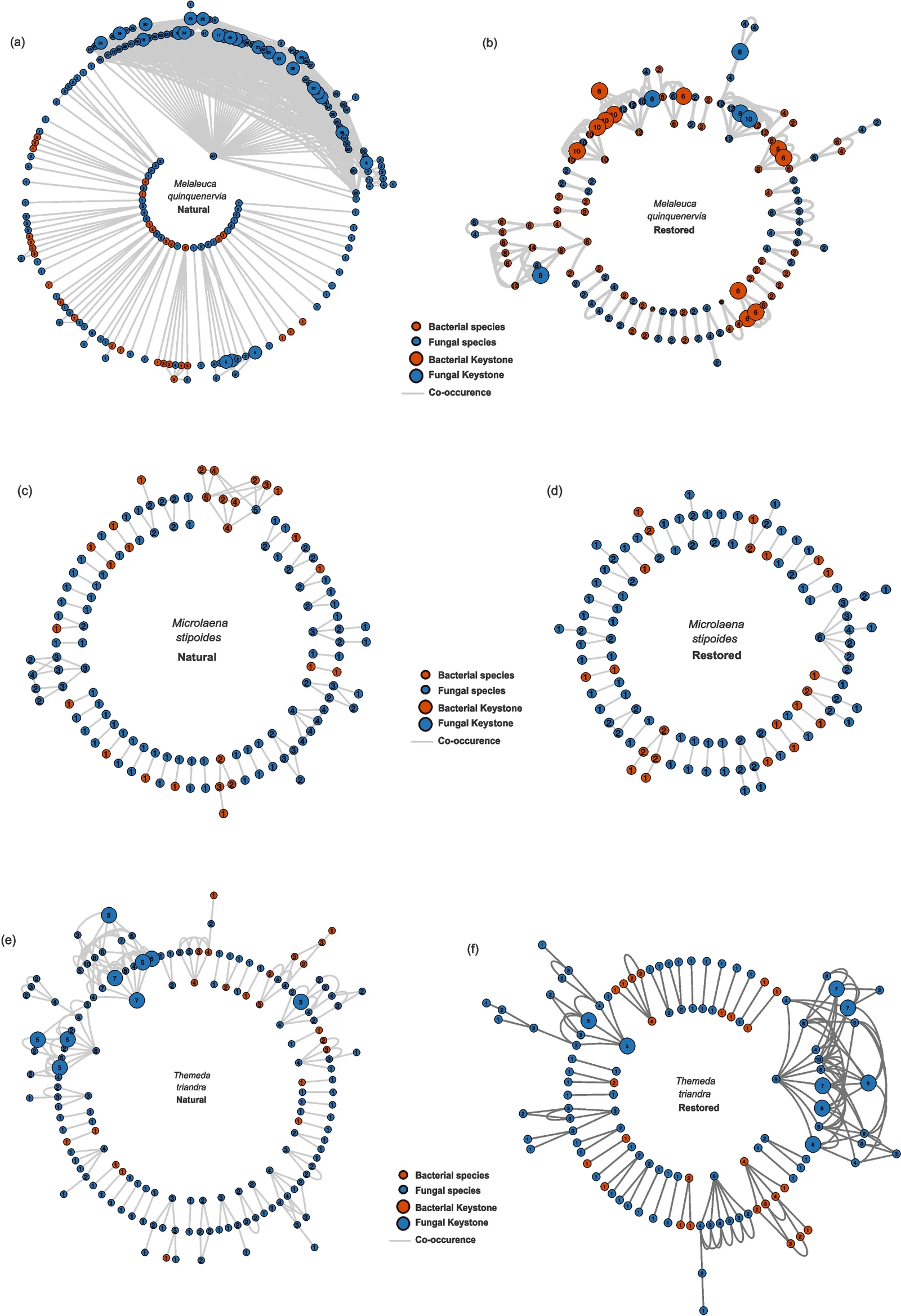
Networks constructed from significant (*p* < 0.05) patterns (shown by grey connecting lines) of co-occurrence between seed bacterial (orange circles) and fungal (blue circles) amplicon sequence variants (ASVs) at restored and natural sites of *Melaleuca quinquenervia* (a, b), *Microlaena stipoides* (c, d) and *Themeda triandra* (e, f). Keystone taxa are identified with increased circle diameter. Numbers on the circles show the number of connections that microbial taxon has with other microbes in the network. Networks constructed in Cytoscape v3.10

The topological attributes of each network, such as degree distribution, mean shortest path and clustering coefficients of the empirical networks were on average different (> 0 average difference) from random networks constructed with an equal number of nodes and edges (Table S7). For *M. quinquenervia* and *M. stipoides* natural networks displayed differences in their structure and topology compared to their respective restored network. (Fig. 7a-d; Table S8a), whereas for *T. triandra* these parameters were similar (Fig. 7e-f)*.* All network connections were of co-occurrence as no significant patterns of mutual exclusion were observed in any of the networks.

The natural networks of *M. quinquenervia* and *M. stipoides* exhibited a greater number of nodes and connections than their restored counterparts (Fig. 7a-d; Table S8b). Moreover, connectivity-related parameters were consistently higher in the natural networks, with significant differences detected in all four parameters for *M. quinquenervia* and in two parameters for *M. stipoides* (Fig. 7c–d; Table S8b). In contrast, although the natural network of *T. triandra* contained more nodes (155 vs. 117) and edges (190 vs. 147) than the restored network, none of the four connectivity parameters differed significantly (Table S8b), indicating comparable levels of ASV connectivity between natural and restored sites for this species.

Across all host species, the highest-scoring clusters (groups of highly connected ASVs) were consistently identified in natural rather than restored networks (Table S8e). The overall top cluster (MCODE score = 11.6) occurred in the *M. quinquenervia* natural network, comprising 24 ASVs connected by 134 edges, all of which were fungal species (Table S8e). In contrast, the leading cluster in the restored *M. quinquenervia* network was smaller, consisting of only four fungal and two bacterial species, and had a substantially lower score (MCODE = 6.0).

### Shared and Keystone ASVs

Very few ASVs were retained in both the restored and natural co-occurrence networks despite a high number of shared ASVs prior to the analyses, for all host species. *Melaleuca quinquenervia* had 213 shared ASVs between the site types prior to analysis, of these only 9.38% were retained in the resultant networks (Table S8j). Of the shared ASVs, four were identified as keystone ASVs in the natural network, although none of these were present in the restored network and a new suite of keystone ASVs were detected. Furthermore, there was a reduction in the number of keystone ASVs from 27 in the natural to 16 in the restored network (Table S8f & g). The top keystone ASVs in the restored network had fewer (10) connections with other ASVs relative to the top keystone within the natural network (56 connections), suggesting that the connectivity of the keystone ASVs within restored sites of *M. quinquenervia* is reduced (Table S8f & g).

*Microlaena stipoides* had 189 shared ASVs in the site types prior to analysis, of these only 7.40% were retained in both the natural and restored networks (Table S8k). No keystone ASVs were identified for *M. stipoides* in either site type. *Themeda triandra* had the highest percentage of shared ASVs (13.5%) retained in both the site type networks (Table S8l). Of these shared ASVs, one keystone ASV identified as *Pestalotiopsis rhizosphorae*, was retained as a keystone in both the natural and restored networks, and in the restored network the connections it has increases from 7 to 9 (Table S8h & i).

### Inter and Intra Kingdom Co-occurrence Patterns

For all hosts, fungal-fungal co-occurrences were the most frequently observed co-occurrences, irrespective of the site type, comprising on average 79.4% (± 9.30 SD) of connections (Fig. 7). Bacterial-bacterial co-occurrences comprised 11.31% (± 6.01 SD) and inter-kingdom bacterial-fungal co-occurrences comprised only 9.28% (± 4.56 SD) of all networks combined. Within *M. quinquenervia*, there was an 11.35% increase in the inter-kingdom interactions within the restored sites, within *M. stipoides* there is a 1.76% decrease, and within *T. triandra*, there is an 8.55% decrease. For each host species the microbial ASV that had the highest number of inter-kingdom interactions (FB interactions) and intra-kingdom (FF or BB interactions) were identified (Table S8c & d). This ASV was always different between the natural and restored sites of each host (Table S8c & d).

## Discussion

Seed-associated microbial communities play a critical but often overlooked role in ecosystem function. Seeds act as dispersal units for both plants and their microbiomes; however, collection, cleaning, and storage of native seed can modify these communities prior to restoration use. Here, we compared seed microbiomes from natural and restored sites to assess whether restored ecosystems host seed-associated microbial assemblages comparable to those of natural reference systems. We found that seed microbiomes differed between natural and restored sites, with the magnitude of divergence varying among host species and between bacterial and fungal communities. Within this system, the seed microbiome of *M. quinquenervia* showed the greatest differentiation between site types, whereas *Themeda triandra* exhibited the highest similarity highlighting host-specific responses to restoration. Our results further support the presence of diverse microbial assemblages within native seeds and show that bacterial taxa associated with restored sites were frequently assigned to functional guilds linked to plant growth promotion.

### Host Species Differ in How Restoration Practices Impact the Seed Microbiome

Host species differed markedly in how restoration influenced seed microbial composition, structure, and network connectivity. *Melaleuca quinquenervia* exhibited the greatest divergence between restored and natural sites, including shifts in taxonomic composition and reduced network connectivity in restored seeds. Core taxa and keystone species present in natural sites were frequently absent from restored-site seeds, particularly within the fungal community and to a lesser extent among bacteria. In contrast, the two grass species showed smaller compositional differences between site types, largely involving less abundant taxa, while core taxa and keystone species were retained. *T. triandra* in particular displayed high similarity in dominant taxa and strong retention of keystone species across restored and natural sites. These patterns suggest that restoration practices involving grasses may better preserve core seed-associated microbes or better allow for post-sowing microbial acquisition processes to reconstruct communities resembling those of natural systems. However, it is important to interpret network-derived “keystone taxa” with caution, as their identification is based on correlation-based inference rather than direct evidence of ecological function and can be sensitive to methodological decisions such as filtering thresholds, network construction parameters, and sample size. We minimised potential bias by applying consistent thresholds and analytical workflows across all comparisons, allowing for robust relative contrasts among host species and site types. Nevertheless, the taxa identified here should be considered as putative keystone candidates rather than confirmed functional hubs in the community. Despite these limitations, network approaches provide a valuable framework for identifying highly connected and potentially influential taxa, offering important hypotheses about microbial community organisation and guiding future experimental validation.

Differences in restoration outcomes may reflect variation in restoration methodologies and seed biology among host species. *M. quinquenervia* requires substantially longer to reach reproductive maturity and produce seed than *M. stipoides* and *T. triandra* [98] potentially contributing to the larger contrasts observed between site types. Restoration approaches also differ, with *M. quinquenervia* commonly established via planted seedlings and grasses typically direct-sown [99], alongside differences in the importance of soil seed banks [100]. The use of tube stock may bypass spermosphere interactions which is a key opportunity for microbial acquisition [101], thereby limiting the establishment of seed microbiomes comparable to those of natural reference sites.

### Restoration and Host Species Differentially Shape Bacterial and Fungal Diversity Recovery

The extent to which restoration altered seed microbial diversity varied among host species and microbial kingdoms. In *M. quinquenervia*, both bacterial and fungal diversity differed significantly between site types but followed contrasting trajectories: bacterial diversity increased in restored sites, while fungal richness declined. Similar kingdom-specific responses to restoration have been reported in soil microbiomes, where bacteria typically recover more rapidly than fungi due to shorter generation times and lower dispersal limitation [102]. Consistent with this, fungal diversity in *M. quinquenervia* seeds of restored sites had not reached levels observed in natural sites at the time of sampling, whereas bacterial diversity exceeded those baselines.

While increased microbial diversity may enhance functional potential and reduce redundancy, contributing to ecosystem stability [103], high microbial richness in degraded systems can also create biotic barriers to recovery by saturating available niches, as observed in post-agricultural restoration contexts [104]. These contrasting interpretations highlight the need to consider both diversity and community composition when evaluating restoration outcomes, and both bacterial and fungal taxa.

In contrast, *M. stipoides* and *T. triandra* did not show divergent bacterial and fungal responses; however, restoration influenced overall diversity differently between the two species. This divergence is notable given that both grasses co-occurred at over half of the sampled sites. Although American grassland studies report rapid convergence of root microbiomes among co-occurring grasses [105], Australian studies have documented strong host-specific differences in microbial recovery following inoculation [106] and restoration [107].

Differences in life history and seed traits may underpin these patterns. *M. stipoides* is a cool-season C3 grass with high seed viability and yield [108], whereas *T. triandra* is a warm-season C4 grass with lower seed viability, frequent sterility, and dormancy requirements [109]. Functional group differences (C3 vs C4) have been shown to influence both bacterial [110] and fungal [111] recruitment in the rhizosphere and may similarly shape the diversity of seed-associated communities.

### Indicator Taxa and Inferred Functional Roles

Indicator species analysis revealed that bacteria strongly associated with restored sites frequently belonged to groups with known plant growth–promoting traits. These included members of the Rhizobiales (e.g. *Lichenihabitans*, *Methylopila*, *Methylobacterium*), as well as *Sphingomonas* and *Paenibacillus*, all of which have been linked to beneficial seed or plant–microbe interactions [112–116]. These taxa were not strongly associated with natural sites across any host species.

Plants in degraded environments may actively recruit beneficial microbes to enhance growth or suppress pathogens [117, 118], suggesting that altered seed microbiomes in restored sites may reflect selection for microbes adapted to nutrient-poor or disturbed conditions. Supporting this, *M. stipoides* has been shown to recruit hydrocarbon-degrading microbes in polluted soils [119] while *T. triandra* alters rhizosphere microbial composition under increasing aridity [120]. Most indicator taxa strongly and significantly associated with restored sites in this study were not core members of the seed microbiome, indicating potential acquisition from the environment following establishment. It is important to note that functional assignments based on taxonomy and database annotations are coarse and do not confirm that these taxa are active, functionally relevant, or performing the same roles in this system. Guild assignments are predictive rather than confirmatory and as such further experimental or functional validation would be required to support these interpretations.

### Native Grass Seeds Share Stable Core Taxa Across Restored and Natural Sites

Across both grass species, three bacterial and two fungal core ASVs were consistently detected across restored and natural sites, accounting for a substantial proportion of microbial diversity, particularly in *T. triandra.* These ASVs closely matched *Pantoea ananatis*, *P. agglomerans*, *Pseudomonas oryzihabitans*, *Alternaria destruens*, and *Cladosporium crousii*. Members of *Pantoea*, *Pseudomonas*, and *Cladosporium* are widely recognised as core seed-associated taxa [121, 122].

Our findings support the concept of a seed microbiome structured around a small, stable core and a larger, more flexible component [123]. The repeated detection of these ASVs across geographically distant sites and contrasting land-use contexts suggests they may play important roles in seed ecology. However, the mechanisms underpinning their persistence remain uncertain. Their widespread occurrence could reflect vertical transmission, consistent environmental acquisition, strong host filtering, or a combination of these processes, and our data cannot distinguish among these alternatives. Additionally, the apparent stability of core taxa across large spatial scales should be interpreted with some caution, as it may be influenced by factors such as detection thresholds, sequencing depth, or the criteria used to define “core” membership.

Despite these uncertainties, the identification of shared core taxa across independent sites provides a useful framework for understanding microbiome assembly and highlights candidate taxa that may contribute disproportionately to host function. These findings offer a foundation for future experimental work aimed at disentangling transmission pathways and testing the functional significance of core seed-associated microbes, as well as informing considerations for maintaining microbiome integrity in ex situ seed collections.

## Conclusion

While soil microbiome studies have demonstrated microbial community shifts associated with restoration, this study shows that seed-associated microbiomes respond in host-specific ways. Differences in seed microbiome diversity, composition, and structure across restored sites suggest that uniform, ecosystem-wide restoration approaches may overlook important host species-level dynamics. In particular, tree species may be less likely to regain seed microbiomes comparable to natural reference systems, potentially due to planting methodologies.

These findings highlight the importance of incorporating microbial perspectives into restoration monitoring, seed collection, and seed storage programs. One application of this knowledge may be for restoration practitioners to consider host-specific strategies to retain microbial diversity, by including seed microbiomes as a complementary approach to soil microbiomes when assessing restoration success and preserving microbial communities during seed handling and storage. Collecting seed from multiple locations and developing protocols to assess microbial diversity in stored seeds may help safeguard the full ecological potential of seeds and their associated microbiomes. These findings suggest that incorporating microbial perspectives into restoration may be valuable, future experimental and mechanistic studies will allow for the research area to determine the functional and ecological significance of the patterns we observed in this study.

## Supplementary Information

Below is the link to the electronic supplementary material.

Supplementary file1 (ZIP 1380 KB)

## Data Availability

Sequences obtained from this study are publicly available in the National Centre for Biotechnology Sequence Read Archive BioProjectID: PRJNA1432314. All analysis code is publicly available at https://github.com/amertin-sudo/Seed-Microbiomes-and-Host-Species and archived on Zenodo (10.5281/zenodo.20281527).
