# Supplementary material for: Host Species Mediate Distinct Seed Microbiome Responses to Restoration
Source: Microb Ecol. 2026 Jun 13;89:155. doi: 10.1007/s00248-026-02802-6 (PMC13461671; doi:10.1007/s00248-026-02802-6)
Supplement: Supplementary file 1 [file 248_2026_2802_MOESM1_ESM.zip › Supplementary_material_v.2.0/Table S5.docx]

| Table S5a. ANOVA summary tables for diversity metric comparisons (Observed ASVs, Simpson's Diversity and Shannon's Diversity) between site types (natural and restored) and host species and pairwise tests between site types for each host species from (a) bacterial or (b) fungal datasets. **Datasets were rarefied to 8,000 and 15,000 reads per sample for the bacterial and fungal datasets, respectively.** | | | | | |  |
| --- | --- | --- | --- | --- | --- | --- |
| **(i) Bacteria - Analysis of Variance Summary Tables** | | | | | | |
| *ANOVA: Observed ASVs Bacteria* |  |  |  |  |  |  |
|  | Df | Sum Sq | Mean Sq | F value | Pr(>F) |  |
| HostSpecies | 2 | 226502 | 113251 | 140.0264 | 2E-16 |  |
| SiteType | 1 | 1168 | 11668 | 2.06 | 0.1535 |  |
| HostSpecies:SiteType | 2 | 3063 | 1532 | 1.89 | 0.1549 |  |
| Residuals | 121 | 97863 | 809 |  |  |  |
| *ANOVA: Simpson’s Diversity Bacteria* |  |  |  |  |  |  |
|  | Df | Sum Sq | Mean Sq | F | Pr(>F) |  |
| HostSpecies | 2 | 3.1793 | 1.589 | 30.2017 | 2.293E-11 |  |
| SiteType | 1 | 0.0051 | 0.00509 | 0.0968 | 0.7563 |  |
| HostSpecies:SiteType | 2 | 0.1452 | 0.07261 | 1.3795 | 0.2556 |  |
| Residuals | 121 | 6.3687 | 0.05263 |  |  |  |
| *ANOVA: Shannon’s Diversity Bacteria* |  |  |  |  |  |  |
|  | Df | Sum Sq | Mean Sq | F value | Pr(>F) |  |
| Source | 2 | 90.175 | 45.088 | 91.6097 | < 2.2e-16 |  |
| SiteType | 1 | 0.165 | 0.165 | 0.3361 | 0.56317 |  |
| Source:SiteType | 2 | 2.384 | 1.192 | 2.4220 | 0.09304 |  |
| Residuals | 121 | 59.553 | 0.492 |  |  |  |
| *Pairwise tests between hosts – Observed ASVs* |  |  |  |  |  |  |
| contrast | estimate | SE | df | t.ratio | p.value |  |
| Melaleuca_quinquenervia-Microlaena_stipoides | 127.92 | 10.50 | 121 | 12.135 | <.0001 |  |
| Melaleuca_quinquenervia-Themeda_triandra | 136.47 | 9.85 | 121 | 13.855 | <.0001 |  |
| Microlaena_stipoides-Themeda_triandra | 8.55 | 6.05 | 121 | 1.413 | 0.3373 |  |
| *Pairwise tests between hosts – Simpson’s Diversity* |  |  |  |  |  |  |
| contrast | estimate | SE | df | t.ratio | p.value |  |
| Melaleuca_quinquenervia-Microlaena_stipoides | 0.311 | 0.0850 | 121 | 3.657 | 0.0011 |  |
| Melaleuca_quinquenervia-Themeda_triandra | 0.491 | 0.0795 | 121 | 6.174 | <.0001 |  |
| Microlaena_stipoides-Themeda_triandra | 0.180 | 0.0488 | 121 | 3.680 | 0.0010 |  |
| *Pairwise tests between hosts – Shannon’s Diversity* |  |  |  |  |  |  |
| contrast | estimate | SE | df | t.ratio | p.value |  |
| Melaleuca_quinquenervia-Microlaena_stipoides | 2.390 | 0.260 | 121 | 9.190 | <.0001 |  |
| Melaleuca_quinquenervia-Themeda_triandra | 2.852 | 0.243 | 121 | 11.736 | <.0001 |  |
| Microlaena_stipoides-Themeda_triandra | 0.462 | 0.149 | 121 | 3.094 | 0.0069 |  |
| **(ii) Fungi - Analysis of Variance Summary Tables** | | | | | |  |
| *ANOVA: Observed ASVs Fungi* |  |  |  |  |  |  |
|  | Df | Sum Sq | Mean Sq | F value | Pr(>F) |  |
| SiteType | 1 | 2615 | 2615 | 3.0723 | 0.080668 |  |
| HostSpecies | 2 | 10037 | 5018.4 | 5.8961 | 0.003082 |  |
| HostSpecies:SiteType | 2 | 52830 | 26414.9 | 31.0347 | 5.76E-13 |  |
| Residuals | 297 | 252788 | 851.1 |  |  |  |
| *Pairwise Tests between site types– Observed ASVs* |  |  |  |  |  |  |
| Melaleuca_quinquenervia |  |  |  |  |  |  |
| contrast | estimate | SE | df | t.ratio | p.value |  |
| Natural - Restored | 37.5 | 6.61 | 297 | 5.67 | <.0001 |  |
| Microlaena_stipoides |  |  |  |  |  |  |
| contrast | estimate | SE | df | t.ratio | p.value |  |
| Natural - Restored | -27.5 | 5.62 | 297 | -4.895 | 0.0540 |  |
| Themeda_triandra |  |  |  |  |  |  |
| contrast | estimate | SE | df | t.ratio | p.value |  |
| Natural - Restored | 16.7 | 5.45 | 297 | 3.061 | 0.0024 |  |
| *ANOVA: Simpson’s Diversity Fungi* |  |  |  |  |  |  |
|  | Df | Sum Sq | Mean Sq | F value | Pr(>F) |  |
| SiteType | 1 | 0.1238 | 0.123819 | 3.4389 | 0.064669 |  |
| HostSpecies | 2 | 0.4122 | 0.20609 | 5.7239 | 0.003638 |  |
| HostSpecies:SiteType | 2 | 0.2771 | 0.138526 | 3.8474 | 0.022406 |  |
| Residuals | 297 | 10.6935 | 0.036005 |  |  |  |
| *Pairwise Tests between site types – Simpson’s* |  |  |  |  |  |  |
| Melaleuca_quinquenervia |  |  |  |  |  |  |
| contrast | estimate | SE | df | t.ratio | p.value |  |
| Natural - Restored | 0.0797 | 0.043 | 297 | 1.854 | 0.0448 |  |
| Microlaena_stipoides |  |  |  |  |  |  |
| contrast | estimate | SE | df | t.ratio | p.value |  |
| Natural - Restored | -0.0384 | 0.0365 | 297 | -1.052 | 0.2937 |  |
| Themeda_triandra |  |  |  |  |  |  |
| contrast | estimate | SE | df | t.ratio | p.value |  |
| Natural - Restored | 0.0931 | 0.0354 | 297 | 2.628 | 0.009 |  |
| *ANOVA: Shannon’s Diversity Fungi* |  |  |  |  |  |  |
|  | Df | Sum Sq | Mean Sq | F value | Pr(>F) |  |
| SiteType | 1 | 2.571 | 2.571 | 5.4196 | 0.0205838 |  |
| HostSpecies | 2 | 8.915 | 4.4573 | 9.3956 | 0.0001105 |  |
| HostSpecies:SiteType | 2 | 11.873 | 5.9365 | 12.5136 | 6.06E-06 |  |
| Residuals | 297 | 140.897 | 0.4744 |  |  |  |
| *Pairwise Tests between site types - Shannon’s* |  |  |  |  |  |  |
| Melaleuca_quinquenervia |  |  |  |  |  |  |
| contrast | estimate | SE | df | t.ratio | p.value |  |
| Natural - Restored | 0.674 | 0.156 | 297 | 4.319 | <.0001 |  |
| Microlaena_stipoides |  |  |  |  |  |  |
| contrast | estimate | SE | df | t.ratio | p.value |  |
| Natural - Restored | -0.307 | 0.133 | 297 | -2.317 | 0.0612 |  |
| Themeda_triandra |  |  |  |  |  |  |
| contrast | estimate | SE | df | t.ratio | p.value |  |
| Natural - Restored | 0.339 | 0.129 | 297 | 2.639 | 0.0088 |  |
| *Pairwise tests between hosts –Observed ASVs* |  |  |  |  |  |  |
| contrast | estimate | SE | df | t.ratio | p.value |  |
| Melaleuca_quinquenervia - Microlaena_stipoides | 12.43 | 4.34 | 297 | 2.866 | 0.0123 |  |
| Melaleuca_quinquenervia - Themeda_triandra | 16.19 | 4.28 | 297 | 3.783 | 0.0005 |  |
| Microlaena_stipoides - Themeda_triandra | 3.77 | 3.91 | 297 | 0.963 | 0.6011 |  |
| *Pairwise tests between hosts – Simpson’s Diversity* |  |  |  |  |  |  |
| contrast | estimate | SE | df | t.ratio | p.value |  |
| Melaleuca_quinquenervia - Microlaena_stipoides | 0.0747 | 0.0282 | 297 | 2.648 | 0.0231 |  |
| Melaleuca_quinquenervia - Themeda_triandra | 0.0934 | 0.0278 | 297 | 3.352 | 0.0026 |  |
| Microlaena_stipoides - Themeda_triandra | 0.0187 | 0.0254 | 297 | 0.733 | 0.7438 |  |
| Pairwise tests between hosts –Shannon’s Diversity |  |  |  |  |  |  |
| contrast | estimate | SE | df | t.ratio | p.value |  |
| Melaleuca_quinquenervia - Microlaena_stipoides | 0.34 | 0.1024 | 297 | 3.321 | 0.0029 |  |
| Melaleuca_quinquenervia - Themeda_triandra | 0.459 | 0.1011 | 297 | 4.542 | <.0001 |  |
| Microlaena_stipoides - Themeda_triandra | 0.119 | 0.0924 | 297 | 1.291 | 0.4016 |  |

| Table S5b. ANOVA summary tables for diversity metric comparisons (Observed ASVs, Simpson's Diversity and Shannon's Diversity) between site types (natural and restored) and host species and pairwise tests between site types for each host species from (a) bacterial or (b) fungal datasets. **Datasets were not rarefied.** | | | | | |  |
| --- | --- | --- | --- | --- | --- | --- |
| **(i) Bacteria - Analysis of Variance Summary Tables** | | | | | | |
| *ANOVA: Observed ASVs Bacteria* |  |  |  |  |  |  |
|  | Df | Sum Sq | Mean Sq | F value | Pr(>F) |  |
| HostSpecies | 2 | 86337 | 43168 | 33.6597 | 5.59E-14 |  |
| SiteType | 1 | 23742 | 23742 | 18.51 | 2.25E-05 |  |
| HostSpecies:SiteType | 2 | 19396 | 9698 | 7.56 | 0.0006196 |  |
| Residuals | 316 | 405268 | 1282 |  |  |  |
| *Pairwise tests between site types* |  |  |  |  |  |  |
| Melaleuca_quinquenervia: |  |  |  |  |  |  |
| contrast | estimate | SE | df | t.ratio | p.value |  |
| Natural - Restored | -38.73 | 7.82 | 324 | -4.950 | <.0001 |  |
| Microlaena_stipoides: |  |  |  |  |  |  |
| contrast | estimate | SE | df | t.ratio | p.value |  |
| Natural - Restored | -19.81 | 6.56 | 324 | -3.020 | 0.0027 |  |
| Themeda_triandra: |  |  |  |  |  |  |
| contrast | estimate | SE | df | t.ratio | p.value |  |
| Natural - Restored | 0.77 | 6.61 | 324 | 0.117 | 0.9073 |  |
| *ANOVA: Simpson’s Diversity Bacteria* |  |  |  |  |  |  |
|  | Df | Sum Sq | Mean Sq | F | Pr(>F) |  |
| HostSpecies | 2 | 4.04 | 2.02 | 34.92 | 1.98E-14 |  |
| SiteType | 1 | 0.47 | 0.47 | 8.24 | 0.0043562 |  |
| HostSpecies:SiteType | 2 | 0.9242 | 0.46210 | 7.979 | 0.0004163 |  |
| Residuals | 316 | 18.30 | 0.05792 |  |  |  |
| *Pairwise tests between site types* |  |  |  |  |  |  |
| Melaleuca_quinquenervia: |  |  |  |  |  |  |
| contrast | estimate | SE | df | t.ratio | p.value |  |
| Natural - Restored | -0.1744 | 0.0526 | 324 | -3.317 | 0.0010 |  |
| Microlaena_stipoides: |  |  |  |  |  |  |
| contrast | estimate | SE | df | t.ratio | p.value |  |
| Natural - Restored | -0.1474 | 0.0441 | 324 | -3.343 | 0.0009 |  |
| Themeda_triandra: |  |  |  |  |  |  |
| contrast | estimate | SE | df | t.ratio | p.value |  |
| Natural - Restored | 0.0633 | 0.0444 | 324 | 1.425 | 0.1551 |  |
| *ANOVA: Shannon’s Diversity Bacteria* |  |  |  |  |  |  |
|  | Df | Sum Sq | Mean Sq | F value | Pr(>F) |  |
| Source | 2 | 113.684 | 56.842 | 75.039 | < 2.2E-16 |  |
| SiteType | 1 | 13.178 | 13.178 | 17.396 | 3.924E-05 |  |
| Source:SiteType | 2 | 18.736 | 9.368 | 12.367 | 6.745E-06 |  |
| Residuals | 316 | 239.371 | 0.758 |  |  |  |
| *Pairwise tests between site types* |  |  |  |  |  |  |
| Melaleuca_quinquenervia: |  |  |  |  |  |  |
| contrast | estimate | SE | df | t.ratio | p.value |  |
| Natural - Restored | -1.041 | 0.190 | 324 | -5.475 | <.0001 |  |
| Microlaena_stipoides: |  |  |  |  |  |  |
| contrast | estimate | SE | df | t.ratio | p.value |  |
| Natural - Restored | -0.529 | 0.159 | 324 | -3.316 | 0.0010 |  |
| Themeda_triandra: |  |  |  |  |  |  |
| contrast | estimate | SE | df | t.ratio | p.value |  |
| Natural - Restored | 0.173 | 0.161 | 324 | 1.076 | 0.2828 |  |
| **(ii) Fungi - Analysis of Variance Summary Tables** | | | | | |  |
| *ANOVA: Observed ASVs Fungi* |  |  |  |  |  |  |
|  | Df | Sum Sq | Mean Sq | F value | Pr(>F) |  |
| SiteType | 1 | 2 | 2 | 0.0014 | 0.96 |  |
| HostSpecies | 2 | 4346 | 2173 | 1.41 | 0.24 |  |
| HostSpecies:SiteType | 2 | 74174 | 37087 | 24.07 | 1.73E-10 |  |
| Residuals | 301 | 509968 | 1541 |  |  |  |
| *Pairwise Tests between site types– Observed ASVs* |  |  |  |  |  |  |
| Melaleuca_quinquenervia |  |  |  |  |  |  |
| contrast | estimate | SE | df | t.ratio | p.value |  |
| Natural - Restored | 20.7 | 7.99 | 301 | 2.591 | 0.01 |  |
| Microlaena_stipoides |  |  |  |  |  |  |
| contrast | estimate | SE | df | t.ratio | p.value |  |
| Natural - Restored | -40.1 | 7.16 | 301 | -5.601 | 0.001 |  |
| Themeda_triandra |  |  |  |  |  |  |
| contrast | estimate | SE | df | t.ratio | p.value |  |
| Natural - Restored | 22.9 | 7.21 | 301 | 3.173 | 0.0016 |  |
| *ANOVA: Simpson’s Diversity Fungi* |  |  |  |  |  |  |
|  | Df | Sum Sq | Mean Sq | F value | Pr(>F) |  |
| SiteType | 1 | 0.1345 | 0.134533 | 3.76 | 0.053 |  |
| HostSpecies | 2 | 0.3568 | 0.178392 | 4.99 | 0.007 |  |
| HostSpecies:SiteType | 2 | 0.2552 | 0.127592 | 3.57 | 0.029 |  |
| Residuals | 301 | 11.8265 | 0.035729 |  |  |  |
| *Pairwise Tests between site types – Simpson’s* |  |  |  |  |  |  |
| Melaleuca_quinquenervia |  |  |  |  |  |  |
| contrast | estimate | SE | df | t.ratio | p.value |  |
| Natural - Restored | 0.0555 | 0.0385 | 301 | 1.441 | 0.015 |  |
| Microlaena_stipoides |  |  |  |  |  |  |
| contrast | estimate | SE | df | t.ratio | p.value |  |
| Natural - Restored | -0.0345 | 0.0345 | 301 | -1.002 | 0.3170 |  |
| Themeda_triandra |  |  |  |  |  |  |
| contrast | estimate | SE | df | t.ratio | p.value |  |
| Natural - Restored | 0.0930 | 0.0347 | 301 | 2.680 | 0.0077 |  |
| *ANOVA: Shannon’s Diversity Fungi* |  |  |  |  |  |  |
|  | Df | Sum Sq | Mean Sq | F value | Pr(>F) |  |
| SiteType | 1 | 2.613 | 2.612 | 5.13 | 0.024 |  |
| HostSpecies | 2 | 6.996 | 3.49 | 6.87 | 0.001 |  |
| HostSpecies:SiteType | 2 | 9.506 | 4.75 | 9.33 | 0.0001 |  |
| Residuals | 301 | 168.458 | 0.50 |  |  |  |
| *Pairwise Tests between site types - Shannon’s* |  |  |  |  |  |  |
| Melaleuca_quinquenervia |  |  |  |  |  |  |
| contrast | estimate | SE | df | t.ratio | p.value |  |
| Natural - Restored | 0.482 | 0.145 | 301 | 3.321 | 0.0010 |  |
| Microlaena_stipoides |  |  |  |  |  |  |
| contrast | estimate | SE | df | t.ratio | p.value |  |
| Natural - Restored | -0.283 | 0.130 | 301 | -2.175 | 0.0563 |  |
| Themeda_triandra |  |  |  |  |  |  |
| contrast | estimate | SE | df | t.ratio | p.value |  |
| Natural - Restored | 0.352 | 0.131 | 301 | 2.690 | 0.0075 |  |
