# Supplementary material for: Host Species Mediate Distinct Seed Microbiome Responses to Restoration
Source: Microb Ecol. 2026 Jun 13;89:155. doi: 10.1007/s00248-026-02802-6 (PMC13461671; doi:10.1007/s00248-026-02802-6)
Supplement: Supplementary file 1 [file 248_2026_2802_MOESM1_ESM.zip › Supplementary_material_v.2.0/Table S8.docx]

| Table S8. Summary tables from network analysis topological parameter comparison, inter-kingdom interactions and keystone species identification analyses. | | | | | | | | | | | |
| --- | --- | --- | --- | --- | --- | --- | --- | --- | --- | --- | --- |
| (a) Network Topology Summary Statistics | | | | | | | | | | | |
| Host | Site type | Starting ASVs | Number of nodes | Number of edges | Avg. number of neighbours | Network diameter | Network radius | Avg. characteristic path length | Clustering coefficient | Network density | Connected components |
| *Melaleuca quinquenervia* | Natural | 521 | 190 | 1142 | 12.021 | 15 | 1 | 2.790 | 0.186 | 0.032 | 32 |
| *Melaleuca quinquenervia* | Restored | 516 | 125 | 316 | 2.528 | 9 | 1 | 2.771 | 0.317 | 0.015 | 33 |
| *Microlaena stipoides* | Natural | 452 | 120 | 101 | 1.683 | 5 | 1 | 1.643 | 0.080 | 0.007 | 42 |
| *Microlaena stipoides* | Restored | 519 | 103 | 72 | 1.398 | 3 | 1 | 1.299 | 0.037 | 0.007 | 36 |
| *Themeda triandra* | Natural | 528 | 155 | 190 | 2.452 | 4 | 1 | 1.518 | 0.117 | 0.008 | 37 |
| *Themeda triandra* | Restored | 433 | 117 | 147 | 2.513 | 4 | 1 | 1.575 | 0.102 | 0.011 | 29 |
| (b) Significance tests between node topological parameters (connectivity related parameters) | | | | | | | | | | | |
| Natural network compared to restored network | Clustering co-efficient \|t\|, | Clustering co efficient p value | Closeness centrality  \|t\| | Closeness centrality p value | Degrees  \|t\| | Degrees  p value | Betweenness centrality  \|t\| | Betweenness centrality p value |  |  |  |
| *Melaleuca quinquenervia* | 4.029144, | <0.0001* | 3.099137 | 0.0025* | 6.44547 | <0.0001* | 0.630247 | 0.5417 |  |  |  |
| *Microlaena stipoides* | 2.14368 | 0.0381* | 0.626 | 0.5311 | 2.42091 | 0.0190* | 0.28446 | 0.7781 |  |  |  |
| *Themeda triandra* | 0.78514 | 0.4370 | 1.060637 | 0.2906 | 0.246074 | 0.8012 | 0.93196 | 0.3602 |  |  |  |

| (c) Network Edge Summary (Fungal-fungal, bacterial-bacterial and intra kingdom connections) | | | | | |
| --- | --- | --- | --- | --- | --- |
| Host | Site type | Number of edges (interactions) | Number of bacterial-bacterial (BB) interactions (%) | ASVs with highest frequency of BB interactions | Closest match in NCBI type sequences database |
| *Melaleuca quinquenervia* | Natural | 1142 | 24  (2.10%) | 138, 108 | *Granulicella acidiphila* (Acidobacteriales), *Brevundimonas albigilva* (Caulobacterales) |
| *Melaleuca quinquenervia* | Restored | 516 | 92 (17.83%) | 11, 169 | *Beijerinckia indica* (Rhizobiales)  *Cellulomonas denverensis* (Micrococcales) |
| *Microlaena stipoides* | Natural | 101 | 18  (17.82%) | 23, 100 | *Pantoea piersonii* (Enterobacterales)  *Kosakonia quasisacchari* (Enterobacterales) |
| *Microlaena stipoides* | Restored | 72 | 9  (12.5%) | 37, 40 | *Rhodopseudomonas pseudopalustris* (Rhizobiales)  *Paenibacillus nuruki* (Paenibacillales) |
| *Themeda triandra* | Natural | 190 | 9  (4.73%) | 38, 50 | *Pantoea ananatis* (Enterobacterales)  *Pantoea ananatis* (Enterobacterales) |
| *Themeda triandra* | Restored | 147 | 19  (12.92%) | 9, 123 | *Acinetobacter guerrae* (Pseudomonadales)  *Curtobacterium oryzae* (Micrococcales) |
| Host | Site type | Number of edges (interactions) | Number of fungal-bacterial (FB) interactions (%) | ASVs with highest frequency of FB interactions | Closest match in NCBI type sequences database |
| *Melaleuca quinquenervia* | Natural | 1142 | 21  (1.83%) | 73, 270 | *Quadrisphaera oryzae* (Kineosporiales)  *Neophaeomoniella eucalypti* (Phaeomoniellales) |
| *Melaleuca quinquenervia* | Restored | 516 | 68 (13.18%) | 240, 320 | *Lichenibacterium ramalinae* (Rhizobiales)  *Cytospora berkeleyi* (Diaporthales) |
| *Microlaena stipoides* | Natural | 101 | 13 (12.87%) | 300 | *Pyrenophora nisikadoi* (Pleosporales) |
| *Microlaena stipoides* | Restored | 72 | 8  (11.11%) | Equal amongst all ASVs | - |
| *Themeda triandra* | Natural | 190 | 24  (12.63%) | 93, 158 | *Phaeoseptoriella zeae* (Pleosporales)  *Diaporthe acaciigena* (Diaporthales) |
| *Themeda triandra* | Restored | 147 | 6  (4.08%) | 31 | *Pantoea piersonii* (Enterobacterales) |
| Host | Site type | Number of edges (interactions) | Number of fungal-fungal (FF) interactions (%) | ASVs with highest frequency of FF interactions | Closest match in NCBI type sequences database |
| *Melaleuca quinquenervia* | Natural | 1142 | 1097 (96.05%) | 347, 370 | *Pseudocastanedospora guangdongensis* (Mycosphaerellales)  *Neovaginatispora aquadulcis* (Pleosporales) |
| *Melaleuca quinquenervia* | Restored | 516 | 356  (68.99%) | 391, 452 | *Xenodevriesia strelitziicola* (Capnodiales)  *Zygosporium pseudogibbum* (Xylariales) |
| *Microlaena stipoides* | Natural | 101 | 70  (69.30%) | 234, 359 | *Eutiarosporella dactylidis* (Botryosphaeriales)  *Epicoccum phragmospora* (Pleosporales) |
| *Microlaena stipoides* | Restored | 72 | 55  (76.38%) | 368, 395 | *Alternaria lawrencei* (Pleosporales)  *Neometulocladosporiella eucalypti* (Helotiales) |
| *Themeda triandra* | Natural | 190 | 157  (82.63%) | 213, 342 | *Cytospora berkeleyi* (Diaporthales)  *Paracamarosporium tamaricis* (Pleosporales) |
| *Themeda triandra* | Restored | 147 | 122  (82.99%) | 198, 282 | *Pestalotiopsis rhizophorae* (Xylariales)  *Aureobasidium xishuangbannaense* (Dothideales) |

| (d) Network Node degree summary | | | | | | |
| --- | --- | --- | --- | --- | --- | --- |
| Host | Site type | Number of nodes | Node with the highest degrees | Number of degrees | Species assigned to node (Closest match in NCBI type sequences database) | Organism type |
| *Melaleuca quinquenervia* | Natural | 190 | 347 | 56 | [*Pseudocastanedospora guangdongensis*](https://www.ncbi.nlm.nih.gov/Taxonomy/Browser/wwwtax.cgi?id=3039569) | Fungi |
| *Melaleuca quinquenervia* | Restored | 125 | 36 | 14 | [*Jatrophihabitans endophyticus*](https://www.ncbi.nlm.nih.gov/Taxonomy/Browser/wwwtax.cgi?id=1206085) | Bacteria |
| *Microlaena stipoides* | Natural | 120 | 300  100 | 5 | *Pyrenophora nisikadoi*  *Kosakonia quasisacchari* | Fungi  Bacteria |
| *Microlaena stipoides* | Restored | 103 | 368 | 6 | *Neofusicoccum microconidium* | Fungi |
| *Themeda triandra* | Natural | 155 | 126 | 10 | *Aureobasidium xishuangbannaense* | Fungi |
| *Themeda triandra* | Restored | 117 | 282 | 10 | *Aureobasidium xishuangbannaense* | Fungi |

| (e) Cluster Analysis summary | | | | | (f) Top cluster metrics | | | |
| --- | --- | --- | --- | --- | --- | --- | --- | --- |
| Host | Site type | Number of nodes | Number of clusters | Total number of nodes that formed clusters | Number of nodes | Number of edges | MCODE Cluster Score (Density x number of nodes) | Organism mix |
| *Melaleuca quinquenervia* | Natural | 190 | 9 | 89 | 24 | 134 | 11.652 | All fungi |
| *Melaleuca quinquenervia* | Restored | 125 | 13 | 54 | 7 | 36 | 6.000 | Five fungi two bacteria |
| *Microlaena stipoides* | Natural | 120 | 7 | 28 | 5 | 10 | 5.000 | All fungi |
| *Microlaena stipoides* | Restored | 103 | 1 | 3 | 3 | 3 | 3.000 | All fungi |
| *Themeda triandra* | Natural | 155 | 10 | 37 | 5 | 10 | 5.000 | All fungi |
| *Themeda triandra* | Restored | 117 | 7 | 24 | 4 | 6 | 4.0 | All fungi |

| (f) *Melaleuca quinquenervia* Natural Sites - Keystone Species | | | | | | | | |
| --- | --- | --- | --- | --- | --- | --- | --- | --- |
| Label | Betweenness Centrality | Closeness Centrality | degree | ASV | Kingdom | Family | Species ID (Closest match in NCBI type sequences database) | Abundance |
| 347 | 0.00 | 0.667 | 56 | fcda6e5477d34187c3354e74411ccccc | Fungi | *Capnodiales* incertae sedis | [*Pseudocastanedospora guangdongensis*](https://www.ncbi.nlm.nih.gov/Taxonomy/Browser/wwwtax.cgi?id=3039569) | 372 |
| 408 | 0.04 | 0.500 | 52 | b1700e7f0e92adc360cb3065cd5be12d | Fungi | *Sporocadaceae* | [*Neopestalotiopsis javaensis*](https://www.ncbi.nlm.nih.gov/Taxonomy/Browser/wwwtax.cgi?id=1562197) | 414 |
| 337 | 0.08 | 0.629 | 49 | e882ba09595916cf30cf2011c0600e9d | Fungi | *Chaetothyriales* incertae sedis | [*Neophaeococcomyces catenatus*](https://www.ncbi.nlm.nih.gov/Taxonomy/Browser/wwwtax.cgi?id=86052) | 371 |
| 469 | 0.04 | 0.545 | 49 | a66f0ef2c13257e1904b0290771dfc94 | Fungi | *Aureobasidiaceae* | *Aureobasidium xishuangbannaense* | 473 |
| 374 | 0.07 | 0.625 | 42 | dd3431bbda24b05a6a3e31d09eba9c20 | Fungi | *Trichomeriaceae* | *Trichomerium wuzetian* | 392 |
| 437 | 0.03 | 0.500 | 34 | 33107f6449484515fc72452f98bcb825 | Fungi | *Capnodiaceae* | *Chaetocapnodium placitae* | 438 |
| 234 | 0.17 | 0.433 | 33 | 4d3cc57da1ade23861ce6acbe70cd5ae | Fungi | *Sympoventuriaceae* | *Pseudosigmoidea alnicola* | 235 |
| 219 | 0.02 | 0.477 | 28 | e5ebe6d6646ab7996c62dece6ddbee80 | Fungi | *Lachnaceae* | *Erioscyphella sasibrevispora* | 220 |
| 403 | 0.16 | 0.600 | 27 | e7f6456d921e809f2d15b343b721d944 | Fungi | *Phacidiales* incertae sedis | *Collophorina rubra* | 404 |
| 322 | 0.04 | 0.600 | 27 | 00ced32aa98362b738d91a3eb9bd195a | Fungi | *Aureobasidiaceae* | *Aureobasidium melanogenum* | 323 |
| 441 | 0.00 | 0.526 | 25 | 56b17ee22867f28d69ceed6536bce3f4 | Fungi | *Nectriaceae* | *Fusarium hainanense* | 442 |
| 427 | 0.01 | 1.000 | 25 | 46e3f5516abff5ba69b0d6309af3b81e | Fungi | *Saccharataceae* | *Saccharata grevilleae* | 428 |
| 432 | 0.00 | 1.000 | 25 | 1df56c2f60a940dd02d5623b7d7d774d | Fungi | *Trichomeriaceae* | *Knufia petricola* | 433 |
| 297 | 0.11 | 0.384 | 25 | 02f5cd858d789688ae9632a4f1bd79a7 | Fungi | *Lachnaceae* | *Erioscyphella papillaris* | 298 |
| 495 | 0.03 | 0.403 | 24 | c5172d4146b1397e88df2c36df0b9b34 | Fungi | *Mycosphaerellaceae* | *Acrodontium crateriforme* | 496 |
| 455 | 0.06 | 0.306 | 21 | 9ca3105a117897b37ca2a3f811964073 | Fungi | *Valsaceae* | *Cytospora heveae* | 456 |
| 497 | 0.05 | 0.295 | 21 | 6d3fe256baa0d15d2e5dfe7cbe6dd8ec | Fungi | *Phyllostictaceae* | *Phyllosticta aristolochiicola* | 498 |
| 363 | 0.06 | 0.667 | 20 | 45e9dbeb4c6f81629d1d1310e078c05d | Fungi | *Hysteriaceae* | *Hysterobrevium walvisbayicola* | 364 |
| 334 | 0.04 | 0.332 | 20 | e3059e979256b4d61c50e91573b08a9b | Fungi | *Lophiostomataceae* | *Anteaglonium queenslandicum* | 335 |
| 433 | 0.05 | 0.390 | 19 | 0c2bf0692b9707596ffe0da601068d31 | Fungi | Verrucariaceae | *Verrucaria modica* | 434 |
| 424 | 0.05 | 0.326 | 18 | 3fa3ad1a16d13ef7591a78511147cb07 | Fungi | Teratosphaeriaceae | *Teratosphaeria corymbiicola* | 425 |
| 309 | 0.01 | 0.343 | 17 | 6f40e983754625c4958310841f7dbd8d | Fungi | *Sporocadaceae* | *Pestalotiopsis grevilleae* | 310 |
| 285 | 0.00 | 0.341 | 6 | 26f7a65a52b2def3ad81a9a584697b55 | Fungi | *Phaeomoniellaceae* | *Nothophaeomoniella ekebergiae* | 318 |
| 407 | 0.13 | 1.000 | 5 | bc91880b91426e5f4bbfb1d788a2d1ab | Fungi | Candelariaceae | *Candelariella ruzgarii* | 549 |
| 489 | 0.00 | 1.000 | 5 | 01a64bb94197f82f4d8dc8d95d54bf79 | Fungi | Teratosphaeriaceae | *Parateratosphaeria persoonii* | 722 |
| 481 | 0.13 | 1.000 | 5 | d55f1a04d1d4b591527d71c1cff3c608 | Fungi | Elsinoaceae | *Elsinoe banksiigena* | 645 |
| 138 | 0.00 | 1.000 | 5 | c30f04f44921b748cd37f91284622d72 | Bacteria | Acidobacteriaceae | *Granulicella acidiphila* | 302 |

| (g) *Melaleuca quinquenervia* Restored Sites - Keystone Species | | | | | | | | |
| --- | --- | --- | --- | --- | --- | --- | --- | --- |
| Label | Betweenness  Centrality | Closeness  Centrality | degree | ASV | Kingdom | Family | Species ID | abundance |
| 242 | 0.12202381 | 0.571428571 | 10 | d66072bb4e903a9c1a95728d29427410 | Bacteria | Microbacteriaceae | *Curtobacterium oryzae* | 371 |
| 233 | 0.136904762 | 0.615384615 | 10 | cca0eab0e34bdd6df5b77dcbcdce88a2 | Bacteria | Sphingomonadaceae | *Sphingosinicella ginsenosidimutans* | 343 |
| 169 | 0.133333333 | 0.625 | 10 | 9cfb89b1a4f17a815dd1c36557343fde | Bacteria | Cellulomonadaceae | *Cellulomonas denverensis* | 9090 |
| 235 | 0 | 0.714285714 | 10 | d0bd24da551a53d14c2d0c7ac2e00d04 | Bacteria | Kineosporiaceae | *Kineosporia aurantiaca* | 5299 |
| 415 | 0.060606061 | 0.571428571 | 10 | 7b6ef155429b51d926b9d917a42c7568 | Fungi | Hypocreaceae | *Trichoderma anaharzianum* | 636 |
| 385 | 0.022727273 | 0.571428571 | 8 | 5bb8c7a5be2027cafc32713a71422e7d | Fungi | Capnodiaceae | *Chaetocapnodium thailandense* | 640 |
| 215 | 0.107142857 | 0.533333333 | 8 | bd4009c04c990b1851da99ca3eb4ec4a | Bacteria | Caulobacteraceae | *Phenylobacterium koreense* | 342 |
| 323 | 0 | 0.368421053 | 8 | 13d714e026ea98d39048d46088bbb27e | Fungi | Cystostereaceae | *Crustomyces albidus* | 2050 |
| 53 | 0.083333333 | 0.714285714 | 6 | 33d9b7db5af91dfb99f537839576474d | Bacteria | Acidobacteriaceae | *Pseudacidobacterium ailaaui* | 165 |
| 165 | 0.05 | 0.555555556 | 6 | 997f0249b100da576e71770c55666efb | Bacteria | Microbacteriaceae | *Curtobacterium gossypii* | 454 |
| 168 | 0 | 0.6 | 6 | 9c4a605f83d83cbe8c3e6db438830fb7 | Bacteria | Acetobacteraceae | *Acetobacteraceae* | 475 |
| 436 | 0.136363636 | 1 | 6 | 936ed71522910d14e4b21ffdedcbcbef | Fungi | Niessliaceae | *Niesslia tenuis* | 593 |
| 155 | 0 | 0.75 | 6 | 9290bf71801644ad69fa09e2d3051852 | Bacteria | Enterobacterales | *Erwinia phyllosphaerae* | 466 |
| 509 | 0 | 0.571428571 | 6 | f895b0acb109f5d3bc1adb5d0360213f | Fungi | Teratosphaeriaceae | *Recurvomyces mirabilis* | 621 |
| 178 | 0 | 0.8 | 6 | a4bdc7ac623bd61af36a78c9b156dcea | Bacteria | Sphingomonadaceae | *Sphingomonas aracearum* | 467 |
| 238 | 0 | 1 | 6 | d3352b6112807e0af3deb9ac0aba0112 | Bacteria | Sphingomonadaceae | *Sphingomonas faucium* | 670 |

| (h) *Themeda triandra* Natural Sites - Keystone Species | | | | | | | | | |
| --- | --- | --- | --- | --- | --- | --- | --- | --- | --- |
| Label | Betweenness  Centrality | Closeness  Centrality | degree | ASV | Kingdom | Family |  | abundance |  |
| 166 | 0.1 | 0.75 | 7 | 184a28d8ee3118f0e6043afdb83eb3ef | Fungi | Pleosporaceae | *Alternaria lawrencei* | 167 |  |
| 204 | 0.17380952 | 0.75 | 7 | 303e9ab3e4d83037ad04ac4dc6ac3750 | Fungi | Sporocadaceae | *Pestalotiopsis rhizophorae* | 215 |  |
| 338 | 0.16746032 | 0.57142857 | 6 | 83e314bd71d4d581997d94e57e0b244b | Fungi | Schizoporaceae | *Xylodon hastifer* | 339 |  |
| 167 | 0 | 0.48275862 | 5 | 19674996b480a65aeb51985dd046e911 | Fungi | Aureobasidiaceae | *Aureobasidium insectorum* | 168 |  |
| 240 | 0 | 1 | 5 | 42cd3aba2a645ef055d779bba22224ce | Fungi | Podonectriaceae | *Podonectria sichuanensis* | 372 |  |
| 248 | 0.16190476 | 0.7 | 5 | 47cd1e4b00d3adad1c071b44e2d8a706 | Fungi | Cladosporiaceae | *Toxicocladosporium strelitziae* | 364 |  |
| 388 | 0.02857143 | 0.41666667 | 5 | a487332fde067e647c157c54ee58673d | Fungi | Cladosporiaceae | *Toxicocladosporium pterocarpi* | 389 |  |
| 280 | 0 | 0.69230769 | 5 | 5aaa7081c74a1cee6e7642ed5cf55813 | Fungi | Capnodiales | *Penidiella aggregata* | 401 |  |
| 378 | 0.03095238 | 1 | 5 | 9a94a02572540c5965d01e4709f5bfca | Fungi | Diaporthaceae | *Diaporthe infecunda* | 493 |  |

| (i) *Themeda triandra* Restored Sites - Keystone Species | | | | | | | | |
| --- | --- | --- | --- | --- | --- | --- | --- | --- |
| Label | Betweenness  Centrality | Closeness  Centrality | degree | ASV | Kingdom | Family | Species ID | abundance |
| 198 | 0.02704678 | 0.625 | 9 | 303e9ab3e4d83037ad04ac4dc6ac3750 | Fungi | Sporocadaceae | *Pestalotiopsis rhizophorae* | 307 |
| 237 | 0.05657895 | 0.66666667 | 7 | 4b1bc7c1a9ba858698ad5018094dd64f | Fungi | Cladosporiaceae | *Cladosporium subcinereum* | 399 |
| 315 | 0.11052632 | 0.66666667 | 7 | 909d5eebd4131699d1148453d74e34d4 | Fungi | Pleosporaceae | *Curvularia coatesiae* | 442 |
| 337 | 0 | 0.5 | 7 | a50bd09cd2e15bbff46f704b39310b26 | Fungi | Phaeosphaeriaceae | *Neostagonospora sorghi* | 445 |
| 178 | 0 | 0.57142857 | 6 | 2166186fb4bd4c395a3515872f995fce | Fungi | Cladosporiaceae | *Cladosporium pini-ponderosae* | 286 |
| 285 | 0.01282051 | 1 | 5 | 766e279d055630cd126f0d21cab99cc1 | Fungi | Naemateliaceae | *Dimennazyma cisti-albidi* | 509 |
| 146 | 0.1025641 | 0.75 | 5 | 05d6331908d55442d11cf0a203d4b757 | Fungi | Pleosporaceae | *Alternaria alstroemeriae* | 288 |
| 321 | 0 | 0.61111111 | 5 | 97b50584cd4ef0ea144145abdc1639e8 | Fungi | Pleosporaceae | *Alternaria destruens* | 518 |

| (j) *Melaleuca quinquenervia* taxa retained in both natural and restored networks | | | |
| --- | --- | --- | --- |
| ASV | Kingdom | Family | Species |
| 45e9dbeb4c6f81629d1d1310e078c05d | Fungi | Hysteriaceae | *Hysterobrevium walvisbayicola* |
| e3059e979256b4d61c50e91573b08a9b | Fungi | Anteagloniaceae | *Anteaglonium queenslandicum* |
| de62ce36cc9535898769812313168bca | Bacteria | Acidobacteriaceae | *Edaphobacter dinghuensis* |
| feeabaccf7f7e8bb81fc625d26d59c45 | Fungi | Teratosphaeriaceae | *Lapidomyces aloidendricola* |
| 5b457d5efc31c2922da5d5e9639606a6 | Fungi | Botryosphaeriaceae | *Diplodia alatafructa* |
| 56b17ee22867f28d69ceed6536bce3f4 | Fungi | Nectriaceae | *Fusarium hainanense* |
| 5ec5151a34c5b6617914f834d1039166 | Fungi | Teichosporaceae | *Teichospora pusilla* |
| 5021c0d27b9c78ded3b2f922c75c3a36 | Fungi | Herpotrichiellaceae | *Capronia kleinmondensis* |
| cd5c89b1e01c2ee418445713c7ef2e1a | Fungi | Nectriaceae | *Microcera larvarum* |
| b339d9bf84b94fee469c60751296f76f | Fungi | Thyridiaceae | *Thyridium curvatum* |
| 353c6311464c88b8e44375a6d403beba | Fungi | Brachybasidiaceae | *Meira nashicola* |
| 9ca3105a117897b37ca2a3f811964073 | Fungi | Valsaceae | *Cytospora heveae* |
| 33dd0ebd99c2b8569ee1921e7eff0623 | Fungi | Stictidaceae | *Cryptodiscus incolor* |
| d1219571cf1bc1f111b62b04756f3980 | Fungi | Xylariaceae | *Occultitheca rosae* |

| (k) *Microlaena stipoides* taxa retained in both natural and restored networks | | | |
| --- | --- | --- | --- |
| ASV | Kingdom | Family | Species |
| 63afe8e6aac58bf0d670a82ca5bc574c | Bacteria | Yersiniaceae | *Rahnella sp.* |
| 53743eace6b0c0d039ca99d32a50e182 | Fungi | Pleosporaceae | *Alternaria lawrencei* |
| d8dfc78f8da44d49ed90b1ab9b0da839 | Fungi | Saccotheciaceae | *Banningia maryelizabethiae* |
| af16991ce12daf846fcbcf604f581dea | Fungi | Apiosporaceae | *Nigrospora camelliae-sinensis* |
| ecbc5a28b2c934708b739622f4b7b4f7 | Fungi | Dothideales incertae sedis | *Selenophoma linicola* |
| 4d2a077848d31fb908c2d6b55a1d3f18 | Fungi | Sporocadaceae | *Bartalinia pondoensis* |
| b4b4ce71117ee941e8230703c663c0b2 | Fungi | Schizoparmaceae | *Coniella paracastaneicola* |
| 3c64c570a53a436ca0372c6d637cc52f | Fungi | Saccotheciaceae | *Aureobasidium xishuangbannaense* |
| 764963d63e736561fbf3e7e7baceb754 | Fungi | Sporocadaceae | *Pestalotiopsis australasiae* |
| 3b94c19ef326eea0918e1c3d244c999c | Bacteria | Erwiniaceae | *Pantoea piersonii* |
| 90e05d3333926ee8560b9c5a503b467c | Fungi | Phaeomoniellaceae | *Neophaeomoniella eucalypti* |
| 57d717789b00b69bc62c3f74e57f03a5 | Bacteria | Kineosporiaceae | *Quadrisphaera oryzae* |
| c7b44c8597bd0404ec91f0dffd8d9339 | Fungi | Sporormiaceae | *Preussia procaviicola* |
| a16fd2ee45187ec576b1c3afc2d277bc | Fungi | Pleosporaceae | *Bipolaris variabilis* |

| (l) *Themeda triandra* taxa retained in both natural and restored networks | | | |
| --- | --- | --- | --- |
| ASV | Kingdom | Family | Species |
| 303e9ab3e4d83037ad04ac4dc6ac3750 | Fungi | Sporocadaceae | *Pestalotiopsis rhizophorae* |
| a85e7d7fb3d4861147f307930b46a96d | Fungi | Teratosphaeriaceae | *Recurvomyces mirabilis* |
| 4b1bc7c1a9ba858698ad5018094dd64f | Fungi | Cladosporiaceae | *Cladosporium subcinereum* |
| 6cf39736ac1f676d72f985394c6685c6 | Fungi | Didymosphaeriaceae | *Pseudopithomyces angolensis* |
| 4297d872e26fb5707dbb9a94b6d7c1c6 | Fungi | Phaeosphaeriaceae | *Phaeosphaeriopsis aloicola* |
| 56b17ee22867f28d69ceed6536bce3f4 | Fungi | Nectriaceae | *Fusarium hainanense* |
| 029e3660a38e2341d1b6316aa02e3ed6 | Fungi | Saccotheciaceae | *Aureobasidium melanogenum* |
| 119b70614392764602bb20f8bbe8f960 | Bacteria | Erwiniaceae | *Pantoea piersonii* |
| ac95b25e6a315d8d49897fcd6fa4f4fc | Fungi | Phaeosphaeriaceae | *Didymocyrtis brachylaenae* |
| 3950057cf81423023446b5dec6aa21f8 | Fungi | Pleosporaceae | *Stemphylium lycopersici* |
| 3ec91b1e27aab6fcc891d8102720eb6a | Fungi | Didymellaceae | *Epicoccum phragmospora* |
| 0462035e2c4ff1b7a9404146ecae7a7b | Fungi | Neoascochyta argentina | *Neoascochyta argentina* |
| 8b0fe00bd6719dfd0b971ce9ea657a06 | Fungi | Bulleribasidiaceae | *Vishniacozyma europaea* |
| 3b94c19ef326eea0918e1c3d244c999c | Bacteria | Erwiniaceae | *Pantoea piersonii* |
| ccd4780ec438e2bafad794ca188d894b | Fungi | Apiosporaceae | *Nigrospora osmanthi* |
| 9685d5efab791e0ab92349203828495c | Fungi | Nectriaceae | *Fusarium robustum* |
| 0908cda00e89f73be7a77ccd53167e3a | Fungi | Apiosporaceae | *Nigrospora macarangae* |
| fb85af020c9c974efe524230ec4c13cd | Fungi | Naemateliaceae | *Dimennazyma cisti-albidi* |
| 7efc9805afda1187988172fd59c54a85 | Fungi | Massarinaceae | *Stagonospora trichophoricola* |
| 610e947f8b18cdd8d9348138281e7096 | Fungi | Quambalariaceae | *Quambalaria rugosae* |
| 476213535665a1076b2c5457d0699e19 | Fungi | Sarocladiaceae | *Sarocladium terricola* |
| 519a47650ee7a90580f77af1816ed81f | Fungi | Periconiaceae | *Periconia imperatae* |
| d3b6d627f63463264786cf9afff3f480 | Fungi | Didymosphaeriaceae | *Paraphaeosphaeria sorghi* |
| 9c6fe943e913c7ed2b88a61d6b5bb7b8 | Fungi | Didymellaceae | *Epicoccum thailandicum* |
| 3acbf9b33248c0a1acf867477a455629 | Fungi | Phaeosphaeriaceae | *Phaeoseptoriella zeae* |
