# Supplementary material for: Host Species Mediate Distinct Seed Microbiome Responses to Restoration
Source: Microb Ecol. 2026 Jun 13;89:155. doi: 10.1007/s00248-026-02802-6 (PMC13461671; doi:10.1007/s00248-026-02802-6)
Supplement: Supplementary file 1 [file 248_2026_2802_MOESM1_ESM.zip › Supplementary_material_v.2.0/Table S4.docx]

| Table S4. Similarity percentages (SIMPER) analysis output detailing species that contributed the most to the similarity between restored and natural sites for each host species.Av. Abund = Average abundance, AV. Sim = Average similarity, Sim SD = similarity standard deviation, Contrib % = percentage contribution to similarity, Cum.% = cumulative contribution of species to similarity. | | | | | | |
| --- | --- | --- | --- | --- | --- | --- |
| **(a) *Melaleuca quinquenervia* – bacterial ASVs** | | | | | | |
| ASV | Bacterial Species | Av.Abund | Av.Sim | Sim/SD | Contrib% | Cum.% |
| f595fccfcd1db925b951905095797142 | *Lichenihabitans psoromatis* | 62.29 | 0.58 | 0.35 | 9.12 | 9.12 |
| b439d77cd049f67634ac7275dbe1c245 | *Lichenibacterium ramalinae* | 31.87 | 0.45 | 0.29 | 7.09 | 16.21 |
| da59261c5663619924bab7e79a737ce6 | *Lichenibacterium minor* | 44.26 | 0.38 | 0.38 | 5.94 | 22.15 |
| 3d6657976bda6c0d850dbfa521a84263 | *Lichenibacterium dinghuense* | 32.52 | 0.35 | 0.4 | 5.53 | 27.68 |
| f8ec009ea7d958c2429fc4d8931f6c25 | *Lichenibacterium ramalinae* | 63.54 | 0.26 | 0.27 | 4.15 | 31.83 |
| 4df385038da1d076640b7c8749be4ae7 | *Lichenicoccus roseus* | 21.61 | 0.21 | 0.19 | 3.35 | 35.18 |
| 745c39aae97a50b799a5a46e126d8c9c | *Amnibacterium setariae* | 96.05 | 0.19 | 0.29 | 3.01 | 38.19 |
| 5e52ed2e74cad7d83f06317b023673e9 | *Lichenibacterium minor* | 38.15 | 0.16 | 0.29 | 2.45 | 40.64 |
| **(b) *Microlaena stipoides* – bacterial ASVs** | | | | | | |
| Bacterial ASV | Species | Av.Abund | Av.Sim | Sim/SD | Contrib% | Cum.% |
| 5673ae66819e3bdbbc09461e4959159e | *Pantoea ananatis* | 2366.25 | 3.45 | 0.42 | 34.6 | 34.6 |
| 428e4a80a1e14a59d72810a32f62f434 | *Pseudomonas oryzihabitans* | 1294.45 | 3.04 | 0.45 | 30.42 | 65.01 |
| cc761daf51f27c423da57f3f1f0ff5cc | *Pantoea agglomerans* | 1168.46 | 1.32 | 0.31 | 13.26 | 78.28 |
| **(c) *Themeda triandra* – bacterial ASVs** | | | | | | |
| Bacterial ASV | Species | Av.Abund | Av.Sim | Sim/SD | Contrib% | Cum.% |
| 5673ae66819e3bdbbc09461e4959159e | *Pantoea ananatis* | 4255.5 | 5.88 | 0.46 | 43.1 | 43.1 |
| cc761daf51f27c423da57f3f1f0ff5cc | *Pantoea agglomerans* | 4920.45 | 5.11 | 0.38 | 37.41 | 80.51 |
| **(d) *Melaleuca quinquenervia* – fungal ASVs** | | | | | | |
| Fungal ASV | Species | Av.Abund | Av.Sim | Sim/SD | Contrib% | Cum.% |
| 13d714e026ea98d39048d46088bbb27e | *Crustomyces albidus* | 4058.27 | 2.98 | 0.33 | 33.18 | 33.18 |
| 303e9ab3e4d83037ad04ac4dc6ac3750 | *Pestalotiopsis rhizophorae* | 1017.29 | 0.98 | 0.29 | 10.97 | 44.15 |
| ab03f1df554dd006f22f5064a432d046 | *Pestalotiopsis grevilleae* | 647.82 | 0.85 | 0.28 | 9.42 | 53.58 |
| 08d6905bddae7391916a2d7dc57bd8ec | *Rosellinia australiensis* | 1238.07 | 0.66 | 0.24 | 7.37 | 60.94 |
| 69c9929bcecd9f63bbe6acf408c17752 | *Diaporthe litchiicola* | 943.38 | 0.5 | 0.24 | 5.61 | 66.55 |
| 764963d63e736561fbf3e7e7baceb754 | *Pestalotiopsis telopeae* | 374.38 | 0.33 | 0.29 | 3.72 | 70.28 |
| 33366dfc8034a4744d5b86e53b34681a | *Pseudocastanedospora guangdongensis* | 280.04 | 0.32 | 0.32 | 3.56 | 73.84 |
| 2166186fb4bd4c395a3515872f995fce | *Cladosporium pseudochalastosporoides* | 200.64 | 0.27 | 0.32 | 2.97 | 76.81 |
| 90e05d3333926ee8560b9c5a503b467c | *Neophaeomoniella eucalypti* | 332.7 | 0.26 | 0.27 | 2.87 | 79.68 |
| 4c785ad0f2935d2c52651ecd7a30e6b6 | *Rosellinia australiensis* | 548.95 | 0.17 | 0.16 | 1.88 | 81.57 |
| **(e) *Microlaena stipoides* – fungal ASVs** | | | | | | |
| Fungal ASV | Species | Av.Abund | Av.Sim | Sim/SD | Contrib% | Cum.% |
| 5e5e52a6f5271a12ce9005e18fc0ef2f | *Alternaria alternata* | 10229.24 | 7.04 | 0.62 | 56.79 | 56.79 |
| 2166186fb4bd4c395a3515872f995fce | *Cladosporium cladosporioides* | 5211.23 | 3.63 | 0.71 | 29.25 | 86.04 |
| **(f) *Themeda triandra* – fungal ASVs** | | | | | | |
| Species |  | Av.Abund | Av.Sim | Sim/SD | Contrib% | Cum.% |
| 5e5e52a6f5271a12ce9005e18fc0ef2f | *Alternaria alternata* | 8756.92 | 8.44 | 0.73 | 52.81 | 52.81 |
| 9c6fe943e913c7ed2b88a61d6b5bb7b8 | *Epicoccum sorghinum* | 7501.89 | 3.45 | 0.38 | 21.6 | 74.4 |
| 2166186fb4bd4c395a3515872f995fce | *Cladosporium cladosporioides* | 2046.06 | 2.24 | 0.74 | 13.99 | 88.39 |
