# Supplementary material for: Host Species Mediate Distinct Seed Microbiome Responses to Restoration
Source: Microb Ecol. 2026 Jun 13;89:155. doi: 10.1007/s00248-026-02802-6 (PMC13461671; doi:10.1007/s00248-026-02802-6)
Supplement: Supplementary file 1 [file 248_2026_2802_MOESM1_ESM.zip › Supplementary_material_v.2.0/Table S3.docx]

Table S3. PerMANOVA summary tables and pairwise comparisons for changes in community composition between site types (natural and restored) and host species and pairwise tests between site types for each host due to a positive interaction term.

| (a) Bacterial communities PERMANOVA, Model = adonis2(formula = bray_dist_matrix ~ Source * SiteType, data = meta, permutations = 999, strata = meta$SitePair) log transformed abundance matrix - Bray Curtis dissimilarities | | | | | | |
| --- | --- | --- | --- | --- | --- | --- |
| Source | Df | SumOfSqs | R2 | F | Pr(>F) |  |
| Host | 2 | 12.969 | 0.0977 | 17.8264 | 0.001 | * |
| SiteType | 1 | 1.939 | 0.01462 | 5.3299 | 0.001 | * |
| HostxSiteType | 2 | 2.794 | 0.02107 | 3.8409 | 0.001 | * |
| Res | 316 | 114.945 | 0.86655 |  |  |  |
| Total | 321 | 132.646 | 1 |  |  |  |
|  |  |  |  |  |  |  |

| (b) Bacterial communities pairwise comparisons between site types | | | | |
| --- | --- | --- | --- | --- |
| combination | SumsOfSqs | F.Model | R2 | P.value.corrected |
| Melaleuca_quinquenervia_Natural <-> Melaleuca_quinquenervia_Restored | 1.719 | 4.145 | 0.04812 | 0.001 |
| Microlaena_stipoides_Natural <-> Microlaena_stipoides_Restored | 1.800 | 4.95 | 0.04029 | 0.001 |
| Themeda_triandra_Natural <-> Themeda_triandra_Restored | 0.914 | 2.762 | 0.02326 | 0.001 |

| (c) Fungal communities PERMANOVA, Model = HostSpecies+SiteType+Host*SiteType = log transformed abundance matrix - Bray Curtis dissimilarities | | | | | | |
| --- | --- | --- | --- | --- | --- | --- |
| Source | Df | SumOfSqs | R2 | F | Pr(>F) |  |
| Host | 2 | 14.207 | 0.1017 | 19.875 | 0.001 | *** |
| SiteType | 1 | 2.337 | 0.01673 | 6.5401 | 0.001 | *** |
| HostxSiteType | 2 | 4.849 | 0.03471 | 6.784 | 0.001 | *** |
| Residual | 331 | 118.3 | 0.84685 |  |  |  |
| Total | 336 | 139.693 | 1 |  |  |  |

| (d) Fungal communities pairwise comparisons between site types | | | | |
| --- | --- | --- | --- | --- |
| combination | SumsOfSqs | F.Model | R2 | P.value.corrected |
| Melaleuca_quinquenervia_Natural <-> Melaleuca_quinquenervia_Restored | 4.62533992 | 14.147 | 0.12961 | 0.001 |
| Microlaena_stipoides_Natural <-> Microlaena_stipoides_Restored | 1.52981706 | 4.037 | 0.03281 | 0.001 |
| Themeda_triandra_Natural <-> Themeda_triandra_Restored | 1.03147268 | 2.863 | 0.02388 | 0.001 |

| (e) Bacterial communities: **PERMANOVA 2, Model = Host+Site+Host*Site = log transformed abundance matrix - Bray Curtis** | | | | | | | |
| --- | --- | --- | --- | --- | --- | --- | --- |
| Source | df | SS | MS | Pseudo-F | P(perm) | Unique perms | Estimates of components of variation (sq. root) |
| Host | 2 | 25697 | 12848 | 4.088 | 0.001 | 998 | 16.484 |
| Site | 32 | 2.75E+05 | 8597.2 | 2.7354 | 0.001 | 992 | 24.194 |
| HostxSi** | 7 | 40792 | 5827.5 | 1.8541 | 0.001 | 996 | 18.396 |
| Res | 318 | 8.80E+05 | 3142.9 |  |  |  |  |
| Total | 321 | 1.33E+06 |  |  |  |  |  |

** Incomplete data as both hosts were not present at each site.

| (f) Fungal communities: **PERMANOVA 2, Model = Host+Site+Host*Site = log transformed abundance matrix - Bray Curtis** | | | | | | | |
| --- | --- | --- | --- | --- | --- | --- | --- |
| Source | df | SS | MS | Pseudo-F | P(perm) | Unique perms | Estimates of components of variation (sq. root) |
| Host | 2 | 35620 | 17810 | 6.2023 | 0.001 | 991 | 19.27 |
| Site | 30 | 3.51E+05 | 11692 | 4.0716 | 0.001 | 981 | 29.117 |
| HostxSi** | 8 | 50606 | 6325.8 | 2.203 | 0.001 | 993 | 20.745 |
| Res | 296 | 8.50E+05 | 2871.5 |  |  |  |  |
| Total | 336 | 1.39E+06 |  |  |  |  |  |

** Incomplete data as both hosts were not present at each site.
