# Supplementary material for: Host Species Mediate Distinct Seed Microbiome Responses to Restoration
Source: Microb Ecol. 2026 Jun 13;89:155. doi: 10.1007/s00248-026-02802-6 (PMC13461671; doi:10.1007/s00248-026-02802-6)
Supplement: Supplementary file 1 [file 248_2026_2802_MOESM1_ESM.zip › Supplementary_material_v.2.0/Table S2.docx]

Table S2. Definitions of network construction parameters used to compare differences in the restored and natural networks of each host species. Definitions are as per the Network Analyser (Assenov et al. 2008).

| **Parameter** | **Definition as per Network Analyser Manual** |
| --- | --- |
| Average Shortest Path length | Average length of a shortest path between *n* and any other node. If *n* is an isolated node, the value of this attribute is zero. |
| Diameter | The diameter is the maximal distance (shortest path) amongst all the distances calculated between each couple of vertexes in graph. The diameter indicates how much distant are the two most distant nodes. It can be a first and simple general parameter of graph "compactness", meaning with that the overall proximity between nodes. A "high" graph diameter indicates that the two nodes determining that diameter are very distant, implying little graph compactness. However, it is possible that two nodes are very distant, thus giving a high graph diameter, but several other nodes are not. Therefore, a graph could have high diameter and still being rather compact or have very compact regions. Notably, the diameter enables to measure the development of a network in time. |
| Degree | The degree is the simplest topological index, corresponding to the number of nodes adjacent to a given node v, where "adjacent" means directly connected. The nodes directly connected to a given node v are also called "first neighbors"  of the given node. Thus, the degree also corresponds to the number of adjacent incident edges. In directed networks we distinguish in-degree, when the edges target the node v, and out-degree, when the edges target the adjacent neighbors of v. |
| Degree Distributions | In undirected networks, the node degree of a node *n* is the number of edges linked to *n*. A self-loop of a node is counted like two edges for the node degree. The node degree distribution gives the number of nodes with degree *k* for *k = 0,1,…*.  In directed networks, the in-degree of a node *n* is the number of incoming edges and the out-degree is the number of outgoing edges. Similar to undirected networks, there are an in-degree distribution and an out-degree distribution. |
| Neighbourhood connectivity | The connectivity of a node is the number of its neighbors. The neighborhood connectivity of a node *n* is defined as the average connectivity of all neighbors of *n*. The neighborhood connectivity distribution gives the average of the neighborhood connectivities of all nodes *n* with *k* neighbors for *k = 0,1,* |
| Network Density | The neighborhood of a given node *n* is the set of its neighbors. The connectivity of *n*, denoted by *k_n_*, is the size of its neighborhood. The average number of neighbors indicates the average connectivity of a node in the network. A normalized version of this parameter is the network density. The density is a value between 0 and 1. It shows how densely the network is populated with edges (self-loops and duplicated edges are ignored). A network which contains no edges and solely isolated nodes has a density of 0. In contrast, the density of a clique is 1. |
| Characteristic Path length | The average shortest path length, also known as the characteristic path length, gives the expected distance between two connected nodes. |
| Clustering Coefficient | In undirected networks, the clustering coefficient *C_n_* of a node *n* is defined as *C_n_* = 2*e_n_*/(*k_n_*(*k_n_*-1)), where *k_n_* is the number of neighbors of *n* and *e_n_* is the number of connected pairs between all neighbors of *n*. In directed networks, the definition is slightly different: *C_n_* = *e_n_*/(*k_n_*(*k_n_*-1)).  In both cases, the clustering coefficient is a ratio *N* / *M*, where *N* is the number of edges between the neighbors of *n*, and *M* is the maximum number of edges that could possibly exist between the neighbors of *n*. The clustering coefficient of a node is always a number between 0 and 1.  The network clustering coefficient is the average of the clustering coefficients for all nodes in the network. Here, nodes with less than two neighbors are assumed to have a clustering coefficient of 0. |
| Connected Components | In undirected networks, two nodes are connected if there is a path of edges between them. Within a network, all nodes that are pairwise connected form a connected component. The number of connected components indicates the connectivity of a network – a lower number of connected components suggests a stronger connectivity. |
| Betweenness centrality | The betweenness centrality *C_b_(n)* of a node *n* is computed as follows: *C_b_*(*n*) = ∑*_s≠n≠t_* (*σ_st_* (*n*) / *σ_st_*), where *s* and *t* are nodes in the network different from *n*, *σ_st_* denotes the number of shortest paths from *s* to *t*, and *σ_st_* (*n*) is the number of shortest paths from *s* to *t* that *n* lies on. Betweenness centrality is computed only for networks that do not contain multiple edges. The betweenness value for each node *n* is normalized by dividing by the number of node pairs excluding *n*: (*N*-1)(*N*-2)*/2*, where *N* is the total number of nodes in the connected component that *n* belongs to. Thus, the betweenness centrality of each node is a number between 0 and 1. The betweenness centrality of a node reflects the amount of control that this node exerts over the interactions of other nodes in the network. This measure favors nodes that join communities (dense subnetworks), rather than nodes that lie inside a community. |
| Closeness centrality | The closeness centrality *C_c_(n)* of a node *n* is defined as the reciprocal of the average shortest path length and is computed as follows: *C_c_*(*n*) = *1* / *avg*( *L*(*n*,*m*) ), where *L*(*n*,*m*) is the length of the shortest path between two nodes *n* and *m*. The closeness centrality of each node is a number between 0 and 1.  NetworkAnalyzer computes the closeness centrality of all nodes and plots it against the number of neighbors. The closeness centrality of isolated nodes is equal to 0.  Closeness centrality is a measure of how fast information spreads from a given node to other reachable nodes in the network. |
