# Supplementary material for: Host Species Mediate Distinct Seed Microbiome Responses to Restoration
Source: Microb Ecol. 2026 Jun 13;89:155. doi: 10.1007/s00248-026-02802-6 (PMC13461671; doi:10.1007/s00248-026-02802-6)
Supplement: Supplementary file 1 [file 248_2026_2802_MOESM1_ESM.zip › Supplementary_material_v.2.0/Table S1.docx]

| Table S1. Site details, plant community type and vegetation form (based on the NSW Vegetation Classification, DPIE, 2020) and location coordinates of restored and natural sites where seed was collected from *Melaleuca quinquenervia*, *Microlaena stipoides* and *Themeda triandra*. NP = national park, NR = nature reserve. | | | | | |  | |  |  |
| --- | --- | --- | --- | --- | --- | --- | --- | --- | --- |
| **Host** | **Site** | **Plant Community Type** | **Vegetation Form** | **Site Type** | **Latitude** | **Longitude** | **Year restoration completed** | **Previous land use history** | **Restoration method** |
| *Melaleuca quinquenervia* | Budgewoi Dune Care | Estuarine Swamp Oak Twig Rush Forest | Forested Wetlands | Restored | -33.23802 | 151.5684755 | 2011 | Sand mining area, rehabilitated using Bitou bush that created a monoculture (now declared a weed). | Tube stock – nursery grown |
|  | Burrawang Reserve, Woy Woy | Umina Coastal Sand Woodland | Dry Sclerophyll Forests (Shrubby sub-formation) | Restored | -33.4981 | 151.3084 | 2007 | Unknown | Tube stock nursery stock. |
|  | Mambo Wetlands | Coastal Sands Swamp Mahogany Rush Forest | Forested Wetlands | Restored | -32.72547 | 152.1040944 | 1990 | Industrial use | Mature plants. Unknown source. |
|  | Novocastrian Reserve | Coastal Sands Swamp Mahogany Rush Forest | Forested Wetlands | Restored | -32.93180 | 151.707976 | Unknown | Unknown | Unknown |
|  | Soldiers Point Park | Coastal Sands Swamp Mahogany Rush Forest | Forested Wetlands | Restored | -32.73604 | 152.0853812 | Unknown | Flood basin. | Unknown |
|  | Colongra Swamp NR | Lower North Creekflat Mahogany Swamp Forest | Forested Wetlands | Natural | -33.20496 | 151.5481722 | - | - | - |
|  | Crowdy Bay NP | Northern Melaleuca quinquenervia Swamp Forest | Forested Wetlands | Natural | -31.69863 | 152.791601 | - | - | - |
|  | Hat Head  NP | Northern Sands Swamp Mahogany Shrubby Rush Forest | Forested Wetlands | Natural | -31.03003 | 153.0250251 | - | - | - |
|  | Lake Innes NR | Northern Melaleuca quinquenervia Swamp Forest | Forested Wetlands | Natural | -31.50224 | 152.8982869 | - | - | - |
|  | Limeburners Creek NP | Coastal Sands Swamp Mahogany Rush Forest | Forested Wetlands | Natural | -31.33082 | 152.9575479 | - | - | - |
|  | Tomaree  NP | Coastal Sands Apple-Blackbutt Forest | Dry Sclerophyll Forests (Shrubby sub-formation) | Natural | -32.74757 | 152.1213281 | - | - | - |
| *Microlaena stipoides* | Blue Gum Point, Yarralumla | - | Grassy Woodlands | Restored | -35.28352 | 149.127762 | 2009 | Pastoral. Then former workman’s Camp “Westlake” in the early 1900s. | Broadcast seeding. |
|  | Burrawang Reserve, Woy Woy | Umina Coastal Sand Woodland | Dry Sclerophyll Forests (Shrubby sub-formation) | Restored | -33.49729 | 151.308359 | 1992 | Sand mining | Unknown |
|  | Crest Reserve, Bankstown | Cumberland Shale Plains Woodland | Grassy Woodlands | Restored | -33.91136 | 150.995836 | 2001 | Sporting Complex | Broadcast seeding |
|  | Elizabeth MacArthur Reserve, Camden | Cumberland Shale Plains Woodland | Grassy Woodlands | Restored | -34.081 | 150.6996 | 2020 | Colonial Farm Estate | Tube stock |
|  | Garrad Reserve, Narrawallee | South Coast Sands Bangalay Forest | Dry Sclerophyll Forests (Shrubby sub-formation) | Restored | -35.3064 | 150.4673 | 1972 | Biodiversity Reserve | Unknown |
|  | Leacock Nature Reserve | Cumberland Shale Plains Woodland | Grassy Woodlands | Natural | -33.9518 | 150.9072 | - | - | - |
|  | Tathra APZ, Panorama Drive | South Coast Foothills Dry Shrub Forest | Dry Sclerophyll Forests (Shrubby sub-formation) | Restored | -36.7239 | 149.9741 | 2019 | Dairy farming land | Locally sourced seed |
|  | Wiseman’s Park, Keiraville | Illawarra Lowland Red Gum Grassy Forest | Grassy Woodlands | Restored | -34.419 | 150.8827 | 2014 | Sporting fields | Broadcast seeding |
|  | Bournda NP North Tura | Far Southeast Headland Scrub | Heathlands | Natural | -36.82828 | 149.935058 | - | - | - |
|  | Jervis Bay NP | Shoalhaven Lowland Bloodwood Shrub Forest | Dry Sclerophyll Forests (Shrubby sub-formation) | Natural | -35.06592 | 150.638115 | - | - | - |
|  | Meroo NP | South Coast Lowland Shrub-Grass Forest | Wet Sclerophyll Forests (Grassy sub-formation) | Natural | -35.3944 | 150.4317 | - | - | - |
|  | Mount Pleasant |  | Grassy Woodlands | Natural | -35.29634 | 149.159899 | - | - | - |
|  | Munmorah SCA | Lower North Coast Headland Clay Heath | Heathlands | Natural | -33.19596 | 151.621238 | - | - | - |
|  | Noorumba Reserve, Rosemeadow | Cumberland Shale Plains Woodland | Grassy Woodlands | Natural | -34.11307 | 150.791854 | - | - | - |
|  | Wyrrabalong North NP | Coastal Sands Apple-Blackbutt Forest | Dry Sclerophyll Forests (Shrubby sub-formation) | Natural | -33.30013 | 151.532938 | - | - | - |
| *Themeda triandra* | Catherine Hill Revegetation Area | Hunter Coast Lowland Apple-Bloodwood Forest | Dry Sclerophyll Forests (Shrubby sub-formation) | Restored | -32.73604 | 152.085412 | 1969 | Coal mining | Mature plants |
|  | Crest Reserve Bankstown | Cumberland Shale Plains Woodland | Grassy Woodlands | Restored | -33.91136 | 150.995836 | 2001 | Sporting Complex | Broadcast seeding |
|  | Elizabeth Macarthur Reserve, Camden | Cumberland Shale Plains Woodland | Grassy Woodlands | Restored | -34.081 | 150.6996 | 2020 | Colonial Farm Estate | Tube stock |
|  | Jesmond Park | Hunter Coast Lowland Spotted Gum Dry Forest | Dry Sclerophyll Forests (Shrubby sub-formation) | Restored | -32.90818 | 151.697962 | 1938 | Soap and tallow works | Unknown |
|  | Norah Head | Coastal Sands Littoral Scrub-Forest | Dry Sclerophyll Forests (Shrubby sub-formation) | Restored | -33.30057 | 151.532811 | 2011 | Gravel quarry | Direct seeding and transplanting of tubestock. |
|  | Short Point Reserve, Merimbula | Far Southeast Headland Scrub | Heathlands | Restored | -36.88462 | 149.932149 | 2016 | Recreational area | Local nursery grown tubestock from local seed and cuttings. |
|  | Tathra APZ, Panorama Drive | South Coast Foothills Dry Shrub Forest | Dry Sclerophyll Forests (Shrubby sub-formation) | Restored | -36.7239 | 149.9741 | 2019 | Dairy farming land | Locally sourced seed |
|  | Yarramundi Grasslands | - | Native Grasslands | Restored | -35.29146 | 149.082151 | 2013 | Sheep grazing | Direct seeding |
|  | Colongra Swamp NR | Hunter Coast Lowland Scribbly Gum Forest | Dry Sclerophyll Forests (Shrubby sub-formation) | Natural | -33.2093 | 151.5501 | - | - | - |
|  | Duesbury Point, Dalmeny | Far Southeast Headland Scrub | Heathlands | Natural | -36.1781 | 150.131368 | - | - | - |
|  | Jervis Bay NP | Shoalhaven Lowland Bloodwood Shrub Forest | Dry Sclerophyll Forests (Shrubby sub-formation) | Natural | -35.0659 | 150.638115 | - | - | - |
|  | Meroo NP | South Coast Lowland Shrub-Grass Forest | Wet Sclerophyll Forests (Grassy sub-formation) | Natural | -35.3944 | 150.4317 | - | - | - |
|  | Mount Pleasant | - | Grassy Woodlands | Natural | -35.2963 | 149.159899 | - | - | - |
|  | Munmorah SCA | Lower North Coast Headland Clay Heath | Heathlands | Natural | -33.1959 | 151.621238 | - | - | - |
|  | Noorumba Reserve, Rosemeadow | Cumberland Shale Plains Woodland | Grassy Woodlands | Natural | -34.1130 | 150.791854 | - | - | - |
