# Supplementary material for: Host Species Mediate Distinct Seed Microbiome Responses to Restoration
Source: Microb Ecol. 2026 Jun 13;89:155. doi: 10.1007/s00248-026-02802-6 (PMC13461671; doi:10.1007/s00248-026-02802-6)
Supplement: Supplementary file 1 [file 248_2026_2802_MOESM1_ESM.zip › Supplementary_material_v.2.0/Figure S1.docx]

Figure S1. Rarefaction curves showing the sequencing depth and number of observed features (amplicon sequence variants, ASVs) of the samples sequenced on the MiSeq Illumina platform for (a) the 16S rRNA gene and (b) the ITS2 region. Each coloured line represents one sample.
