# Supplementary material for: Host Species Mediate Distinct Seed Microbiome Responses to Restoration
Source: Microb Ecol. 2026 Jun 13;89:155. doi: 10.1007/s00248-026-02802-6 (PMC13461671; doi:10.1007/s00248-026-02802-6)
Supplement: Supplementary file 1 [file 248_2026_2802_MOESM1_ESM.zip › Supplementary_material_v.2.0/Table S7.docx]

| Table S7. Average differences between the random and empirical networks, and differences in five network topological parameters | | | | | | | |
| --- | --- | --- | --- | --- | --- | --- | --- |
| Host | Network Type | Average difference | Difference in shortest path length | Difference in Clustering Coefficient | Difference in Closeness Centrality | Difference in Betweenness Centrality | Difference in Neighbourhood Connectivity |
| *Melaleuca quinquenervia* | Natural | 0.35205 | 0.67895 | 0.32632 | 0.40000 | 0.33684 | 0.59474 |
|  | Restored | 0.65600 | 0.82400 | 0.46400 | 0.67200 | 0.57600 | 0.78400 |
| *Microlaena stipoides* | Natural | 0.30370 | 0.65000 | 0.20000 | 0.38333 | 0.27500 | 0.30000 |
|  | Restored | 0.22546 | 0.47573 | 0.09709 | 0.44660 | 0.21359 | 0.11650 |
| *Themeda*  *triandra* | Natural | 0.43584 | 0.92258 | 0.36774 | 0.54194 | 0.30323 | 0.30323 |
|  | Restored | 0.41785 | 0.89744 | 0.32479 | 0.57265 | 0.24786 | 0.35043 |
