# Supplementary material for: Host Species Mediate Distinct Seed Microbiome Responses to Restoration
Source: Microb Ecol. 2026 Jun 13;89:155. doi: 10.1007/s00248-026-02802-6 (PMC13461671; doi:10.1007/s00248-026-02802-6)
Supplement: Supplementary file 1 [file 248_2026_2802_MOESM1_ESM.zip › Supplementary_material_v.2.0/Supplementary Results.docx]

### *Data quality control and accuracy and completeness of taxonomy assignment*

The *decontam* analysis of 16S data identified eighty-one bacterial extraction contaminants and fifty-four PCR bacterial contaminants comprising 7.797% and 2.97% of the dataset, respectively (Table 1). After removal of contaminant amplicon sequence variants (ASVs), and the control and mock community samples a total of 3,148 ASVs were identified from 322 samples. The *decontam* analysis of ITS2 data identified eight fungal extraction contaminants and five PCR fungal contaminants comprising 1.305% and 1.133% of the dataset, respectively (Table 1). Upon inspection of the taxonomic identity of the contaminants, and upon consideration of the small percentage of the dataset that they comprise, these ASVs were removed from further analyses.

Assignment of taxonomy to the bacterial ASVs resulted in 99.42% of reads being assigned to a phylum, 95.73% to a family, and 88.38% to a genus. Confidence levels (ranging from 0-1) from the taxonomic assignment had an average value of 0.94, max = 1.0, min = 0.40). Assignment of taxonomy to the fungal ASVs resulted in 100% of reads being assigned to a phylum, 94.03% to a family, and 86.14% to a genus. Confidence levels (ranging from 0-1) from the taxonomic assignment had an average value of 0.92, max = 1.0, min = 0.70).

### *Mock community, technical replicate and sequencing run variation*

Mock community bacterial and fungal controls showed that for each sequencing run all taxa that were present in the mock community were retrieved. For the bacteria, the relative abundance of *Erwinia aphidicola* differed between the first and third sequencing runs (ANOVA_Asv*Run_ F = 2.53, d.f. = 6,37, p = 0.045), whilst all other taxa showed no significant difference in abundance between runs (Figure 2a). The highest variation between technical replicates for the mock bacterial samples was seen in run three where technical replicates saw a variation of 3,216 reads of *Agrobacterium larrymoorei* (Table 2a)*.*

There was no significant difference in the relative abundance of each fungal species in the fungal mock community between the three sequencing runs (ANOVA_Asv*Run_ F = 1.76, d.f. = 10,47, p = 0.1117) (Figure 2b). For the fungal community the highest variation between technical replicates was seen in run two with a variation of 3,372 reads between replicates of *Schizophyllum commune* (Table 2b). These results suggest that the majority of the microbial community estimates did not differ significantly between sequencing runs or technical replicates.

| Table 1. Most prevalent amplicon sequence variants (> 0.01 % of dataset) detected in the negative control samples (i.e. blank DNA extractions and no-template PCRs) and the proportion of the dataset they comprise based on outputs from the prevalence method in the R package ‘decontam’ (Davis et al., 2018) with p = 0.1. | | | | | | |
| --- | --- | --- | --- | --- | --- | --- |
| (a) **Extraction contaminants (bacteria)** | | | | | | |
| ASV Identifier | Family | Genus | | Proportion of dataset | |  |
| 945184b6386c192c0066e0a98a154780 | Enterobacteriaceae | *Erwinia* | | 3.81614382 | |  |
| 7521edd61e578d0d49cfd01fbf8cc2f5 | Xanthomonadaceae | *Xanthomonas* | | 1.56139672 | |  |
| db71e335bc263a02d10d6331d567e4ba | Xanthomonadaceae | *Stenotrophomonas* | | 0.70414012 | |  |
| 5648dccee530d68ceb3e4d7d22cf8756 | Pseudomonadaceae | *Pseudomonas* | | 0.60353028 | |  |
| 9bb276530d60c2d320b7fd8bc8cf8613 | Comamonadaceae | *Comamonadaceae* | | 0.28372644 | |  |
| fd1098cd571337c869bc804919b0f630 | Bacillales | *Bacillales* | | 0.21105663 | |  |
| 06f825b512d903b9230e1a55d87359ee | Streptococcaceae | *Streptococcus* | | 0.15149416 | |  |
| 50c1adc394d69456bab0180bd2d25d57 | Chitinophagaceae | *Sediminibacterium* | | 0.10604334 | |  |
| 65d43491988bfe557da4d86a5ba25dae | Staphylococcaceae | *Staphylococcus* | | 0.06412043 | |  |
| 85c44c83eddc5d3028261a1000b7d0e1 | Gemellaceae | *Gemella* | | 0.04658388 | |  |
| 61d96b86c674ec6276984c9be7a81efc | Sphingomonadaceae | *Novosphingobium* | | 0.03749371 | |  |
| a8e53bd66879cd51ecb4e7daf552accb | Bifidobacteriaceae | *Gardnerella* | | 0.03023188 | |  |
| b1a2eb74f17971864ed1ed964da108e5 | Leptotrichiaceae | *Sneathia* | | 0.01722754 | |  |
| 1444c4f113d87be26e293a001d270dcc | Burkholderiaceae | *Burkholderia* | | 0.01699578 | |  |
| cd9401a6bce4a63af516d06d2a843f9d | Veillonellaceae | *Veillonella* | | 0.01272108 | |  |
| ca9c66d47347c03d0343d6fe03ed8636 | Pasteurellaceae | *Haemophilus* | | 0.0120773 | |  |
| e96a18c7e3d537fc5fcb8f8ed70414c9 | Xanthobacteraceae | *Xanthobacteraceae* | | 0.01158803 | |  |
| d829bee4984f82ffc2453212157caf96 | Xanthobacteraceae | *Bradyrhizobium* | | 0.01151078 | |  |
| (b) **PCR contaminants: Bacteria** | | | | | | |
| bdf8a26094624622d68509a87fa75ba7 | Bacillaceae | *Bacillus* | | 2.33104637 | |  |
| fd98346394a5c79e554003012cb33826 | Micrococcaceae | *Micrococcaceae* | | 0.36662335 | |  |
| 0c579d21280801f02a641e1608606927 | Micrococcaceae | *Micrococcus* | | 0.0597615 | |  |
| d6efd2da2728fd74ded268122ee05036 | Sphingomonadaceae | *Sphingomonas* | | 0.03117343 | |  |
| 820f6693f569e339f183638cd73a7fe6 | Corynebacteriaceae | *Lawsonella* | | 0.02374053 | |  |
| 9908fffab7ed4f3bec44cda2f5084d49 | Enterococcaceae | *Enterococcus* | | 0.02115517 | |  |
| 0dbf680e00aa85eb226b129f0adeff7e | Dermacoccaceae | *Dermacoccus* | | 0.01871897 | |  |
| 11397adbcc3ae7ea2d586775e298a19d | Sphingomonadaceae | *Sphingomonas* | | 0.01655622 | |  |
| 8612a638846acf832935cb932a1f0398 | Weeksellaceae | *Cloacibacterium* | | 0.01655622 | |  |
| 958d54f6c2724fdc04ad01d39d59aaca | Prevotellaceae | *Prevotella* | | 0.01583531 | |  |
| 5a7b179b1b45f0fe2282f260bf073f60 | Propionibacteriaceae | *Cutibacterium* | | 0.01581045 | |  |
| (c) **Extraction contaminants: Fungi** | | | | | | |
| 2531d4fe544b32d7f0da1bdc3e99b794 | Pleosporaceae | *Alternaria* | | 0.71946897 | |  |
| 30fd7c708b42567f17ee98c3ba178b7b | Phleogenaceae | *Atractidochium* | | 0.37184598 | |  |
| 997cabda991ca083984f05c60b63350e | Saccotheciaceae | *Aureobasidium* | | 0.20440756 | |  |
| (d) **PCR contaminants: Fungi** | | | | | | |
| 1334cef728f62bc9ce09ee6dc98a25ac | Ceratobasidiaceae | |  | | 0.56863404 | |
| cedaf0ee87219d214baa5139121c1a01 | Plectosphaerellaceae | *Chordomyces* | | 0.55624401 | |  |

Figure 2a. Compound bar plot showing relative abundances of the bacterial amplicon sequence variants within the mock community constructed to test for batch effects of the multiple Illumina sequencing runs. Different columns show the three technical replicates used per sequencing run for each of the three runs.

Figure 2b. Compound bar plots showing relative abundances of the fungal amplicon sequence variants within the mock community constructed to test for batch effects of the multiple Illumina sequencing runs. Different columns show the three technical replicates used per sequencing run for each of the three runs.

Table 2. Read count variation (number of post QC reads plus standard deviation) amongst technical replicates constructed from a mock community comprised of either (a) six bacterial species or (b) seven fungal species. Technical replicates were constructed for each of the three sequencing runs undertaken in this study (Run1, Run2 and Run3).

| (a) **Bacterial species** | **Run1**  **Replicate1** | **Run1**  **Replicate2** | **Run1**  **Replicate3** | **Run2**  **Replicate1** | **Run2**  **Replicate2** | **Run2**  **Replicate3** | **Run3**  **Replicate1** | **Run3**  **Replicate2** | **Run3**  **Replicate3** | **Standard deviation**  **Run1** | **Standard deviation**  **Run2** | **Standard deviation**  **Run3** |
| --- | --- | --- | --- | --- | --- | --- | --- | --- | --- | --- | --- | --- |
| *Pantoea eucalyptii* | 15984 | 19884 | 19545 | 18806 | 17547 | 13147 | 21569 | 25328 | 25223 | 2160 | 2971 | 2140 |
| *Agrobacterium larrymoorei* | 0 | 0 | 0 | 0 | 0 | 0 | 27332 | 33556 | 31855 | 0 | 0 | 3216 |
| *Pseudomonas rhodesiae* | 8309 | 12525 | 9477 | 7317 | 6840 | 9924 | 4840 | 5437 | 6199 | 2176 | 1660 | 681 |
| *Erwinia aphiciola* | 6136 | 3159 | 4919 | 2620 | 2780 | 4577 | 10282 | 12816 | 14513 | 1496 | 1086 | 2129 |
| *Curtobacterium herbarum* | 444 | 400 | 417 | 1118 | 1056 | 1542 | 802 | 834 | 907 | 22 | 264 | 53 |
| *Bacillus proteolyticus* | 0 | 0 | 0 | 0 | 0 | 0 | 2510 | 2688 | 2928 | 0 | 0 | 209 |

| **(b) Fungal species** | **Run1**  **Replicate1** | **Run1**  **Replicate2** | **Run1**  **Replicate3** | **Run2**  **Replicate1** | **Run2**  **Replicate2** | **Run2**  **Replicate3** | **Run3**  **Replicate1** | **Run3**  **Replicate2** | **Run3**  **Replicate3** | **Standard deviation**  **Run1** | **Standard deviation**  **Run2** |
| --- | --- | --- | --- | --- | --- | --- | --- | --- | --- | --- | --- |
| *Schizophyllum commune* | 6488 | 8826 | 8924 | 10813 | 8576 | 6332 | 7495 | 8099 | 2811 | 3372 | 898 |
| *Alternaria alternata* | 5696 | 7981 | 8584 | 9445 | 8263 | 5755 | 5287 | 7752 | 2395 | 2876 | 1309 |
| *Fusarium equiseti* | 5037 | 5552 | 6326 | 6451 | 7462 | 5062 | 3308 | 6584 | 1914 | 2328 | 1639 |
| *Cladosporium augustifolium* | 3912 | 5577 | 5088 | 6442 | 5223 | 4014 | 5653 | 5012 | 1571 | 1893 | 825 |
| *Diaporthe hongkongensis* | 3327 | 4460 | 4719 | 5544 | 4635 | 3332 | 4152 | 4585 | 1218 | 1475 | 636 |
| *Clonostachys rosea* | 2801 | 4125 | 3739 | 4676 | 3861 | 2842 | 4138 | 3601 | 831 | 1000 | 651 |
| *Cylindriobasidium eucalypti* | 885 | 1102 | 1077 | 1517 | 1127 | 838 | 2268 | 1056 | 231 | 302 | 770 |
