# Supplementary material for: Host Species Mediate Distinct Seed Microbiome Responses to Restoration
Source: Microb Ecol. 2026 Jun 13;89:155. doi: 10.1007/s00248-026-02802-6 (PMC13461671; doi:10.1007/s00248-026-02802-6)
Supplement: Supplementary file 1 [file 248_2026_2802_MOESM1_ESM.zip › Supplementary_material_v.2.0/Table S6.docx]

| Table S6. Amplicon sequence variants identified as indicator species, the associated IndVal statistic and the associated significance level (p value) for At = 0.2 and Bt = 0.2. Results are reported separately for each site type and host species. | | |
| --- | --- | --- |
| (a) **Melaleuca quinquenervia - Bacterial** |  |  |
| **Group Natural** |  |  |
| **ASV** | **IndVal statistic** | **p value** |
| 4df385038da1d076640b7c8749be4ae7 | 0.644 | 0.004 |
| 1e30c80a316e14620f2c4167ace27cf1 | 0.592 | 0.002 |
| d193cc6097df8ae99117409f070707ad | 0.379 | 0.031 |
| 8f37e23172b156b9ec457962f5eb03d4 | 0.354 | 0.015 |
| cc5231e86ab16bebc2a59cf7caa9ea8b | 0.354 | 0.018 |
| **Group Restored** |  |  |
| **ASV** | **IndVal statistic** | **p value** |
| da59261c5663619924bab7e79a737ce6 | 0.826 | 0.001 |
| 913b41de2d1b26b57c95a2eca092c1b9 | 0.783 | 0.001 |
| 3d6657976bda6c0d850dbfa521a84263 | 0.77 | 0.001 |
| 60a8843517f35173407a9d146488e1ac | 0.692 | 0.001 |
| 04ecfad5772d2e09a84a0f5ef460536c | 0.684 | 0.003 |
| af6dae6d1cfbd2b67ba39e01196dc488 | 0.662 | 0.001 |
| 31e15e64c03b448bc198a28b085a813a | 0.627 | 0.001 |
| 38f00d1309a0f4feb0d7abbb06eef61f | 0.624 | 0.002 |
| e95b4277f9caf56d3660e6b3d08eec7d | 0.622 | 0.001 |
| c57740c1835bc24dd31f70a8b6b4c25d | 0.619 | 0.001 |
| d66072bb4e903a9c1a95728d29427410 | 0.619 | 0.001 |
| 9836f999b9346c340d6190675a1d4687 | 0.603 | 0.001 |
| d9e27f72e0d856ea7072e4ef41eb554b | 0.603 | 0.001 |
| 095d4f88b91691c3e0dc2ed7b4054739 | 0.584 | 0.001 |
| f097eca1f256195f7b815c0eeb66b88f | 0.584 | 0.001 |
| c3636e623bf4cb134b4315911e2f2553 | 0.576 | 0.003 |
| 453cf07d3e96e304f23fbde7b355fe70 | 0.574 | 0.004 |
| 8799545e4f02b51ebc2b9ea302d8af4a | 0.57 | 0.005 |
| 1ec9fd06c88669eb15764e0b441ade46 | 0.565 | 0.011 |
| dc662c882f3f607acaf637da47e69781 | 0.564 | 0.002 |
| ee0c3620e3e4a9f2b2dc854c55abad85 | 0.564 | 0.001 |
| 497a26126c9e726c768bdec79e8c4e9b | 0.564 | 0.001 |
| 46edd9a681a1c394007859666f76d477 | 0.559 | 0.001 |
| e24e1799231b926cd90ce8f7e8dd2dd4 | 0.553 | 0.005 |
| 90191597459e307ec25618c20b93ada6 | 0.549 | 0.018 |
| 26f173c04574871aba8876838e2d9e61 | 0.54 | 0.001 |
| a9164d01308501b576afa06256a15b0f | 0.54 | 0.002 |
| 29115e7bd8ef43044853d0193cf6f622 | 0.536 | 0.001 |
| e35dbd7c50f8902e535eefbb222eb7be | 0.528 | 0.032 |
| e4a9c1dc7739105bfbd086ec4faf137a | 0.51 | 0.004 |
| 99bd44b548fbcc1d18e7f9525484c1ec | 0.499 | 0.01 |
| aa6972ccc566f0de70e4d76e0ab18026 | 0.493 | 0.005 |
| 3e7e79afb6b6c4c37d06edb97bc7f4fa | 0.486 | 0.013 |
| 2e32a0610d3bb415126655fbc793b655 | 0.479 | 0.019 |
| e7ddf233bed17461088aae76cddd444e | 0.477 | 0.003 |
| ea50ab99242e77fd8d897c2c43449f84 | 0.477 | 0.002 |
| 1c2b8892a0d7f828d72808cd01cfd199 | 0.452 | 0.004 |
| 22c2271f833dfa1dfc209cfa8fdfeba6 | 0.452 | 0.004 |
| 7b3fd798bc4d1a3b51cd9055f7ae1ce7 | 0.452 | 0.001 |
| 16c0e3b150b4876a0a40172021480cb1 | 0.437 | 0.024 |
| ae04dca2a07ec1aa560e836bc399ea57 | 0.433 | 0.023 |
| 49543652ce5c14740218e67e9697a454 | 0.426 | 0.009 |
| 6658bf5f4046896bfa595c0ce549115e | 0.426 | 0.011 |
| b449f5066387be07ee577a95585d45ca | 0.426 | 0.007 |
| 884726d2c6c673f65a53b9e1ced78005 | 0.399 | 0.021 |
| 9914784b1470f7d2d4116d75c6afce03 | 0.399 | 0.01 |
| b867276ef61fdee848c1d4b268640323 | 0.399 | 0.012 |
| 66bd946c2bf0670e6faa4b5231689abf | 0.398 | 0.018 |
| 2a39f0b75d31d00b3136b2edbe82962e | 0.384 | 0.044 |
| e30486048d5e00f97cc26969618a161a | 0.374 | 0.045 |
| 0514504356ad4964fd4c29b13370226d | 0.369 | 0.025 |
| 38ff6a0d927575e1d4dfc646197d5eac | 0.369 | 0.038 |
| 889f1bc31887efea2354ba099342a203 | 0.369 | 0.026 |
| 90abd727c9199a3dd21e3c38b3dff0c3 | 0.369 | 0.03 |
| 997f0249b100da576e71770c55666efb | 0.369 | 0.031 |
| a0901407705992dc213ac509ede97d47 | 0.369 | 0.027 |
| b01cdc53ccf0147bfd6e571cbe2af6b6 | 0.369 | 0.034 |
| b92f9902497d8097a399dd069f2e5a57 | 0.369 | 0.029 |
| de7af049a77747324787958d904175ab | 0.369 | 0.024 |
|  |  |  |
| (b) **Microlaena stipoides - Bacterial** |  |  |
| **Group Natural** |  |  |
| **ASV** | **IndVal statistic** | **p value** |
| d15bc449222795a9ff230013aa633686 | 0.671 | 0.001 |
| dcba105f35d8ebc9e22269c7491ad3a7 | 0.357 | 0.005 |
| 3ef3bf00702397006fafc0fab1ba9d93 | 0.319 | 0.032 |
|  |  |  |
| **Group Restored** |  |  |
| **ASV** | **IndVal statistic** | **p value** |
| f0a2f6acf476be1b31b832b83529d6c5 | 0.691 | 0.001 |
| e1c7d97fe13e9127d225d76a1feb8c78 | 0.684 | 0.003 |
| 166093520348b8c5ef6a4eb79faae728 | 0.675 | 0.001 |
| c22b16cc6108c04f29fea3b6d4c81571 | 0.668 | 0.001 |
| 04ecfad5772d2e09a84a0f5ef460536c | 0.618 | 0.011 |
| c06ccd1f7d57566ef669942328b1a946 | 0.616 | 0.001 |
| 019dbae2126663ddb0710cfa00fccb48 | 0.588 | 0.003 |
| 10afda2baef44de4c584a6641de399b1 | 0.554 | 0.001 |
| 3d5baff45729bf2214074c2e9f77ad10 | 0.541 | 0.001 |
| 893f998a57f8d7782182a7aa1cb8eeed | 0.511 | 0.001 |
| 45e3696d78d57fc35094a6cf67e6414b | 0.509 | 0.002 |
| 146195f72933e590d98b89c38d6eda33 | 0.506 | 0.006 |
| a0901407705992dc213ac509ede97d47 | 0.493 | 0.013 |
| 8df2b2e6c1cb64bd18b81d5bae7f0898 | 0.469 | 0.021 |
| 3ebe761bfb1238c87195d431f41bf976 | 0.446 | 0.004 |
| 57d717789b00b69bc62c3f74e57f03a5 | 0.425 | 0.047 |
| d0ec34dbfb350b13b941d51df55d392e | 0.424 | 0.011 |
| f8aeec3c1ff0f4e7e1c829e4db0618d9 | 0.398 | 0.023 |
| 2d77cf80c9b03b82ce7ed6eacfa04e12 | 0.392 | 0.004 |
| a8377fd1db22cfbfa2ceb1d14d392dc2 | 0.389 | 0.008 |
| 605ac9a7e88d47be1bf40c55de175d79 | 0.372 | 0.016 |
| a76e065bdfde0aa883c898ccf5f092fc | 0.372 | 0.016 |
| 397b2f8e42f9fa4f7e37216716ebfcfe | 0.351 | 0.029 |
| 4608fae4bbc9964cdd17af8782f2155e | 0.351 | 0.027 |
| 708e50f6b07a7fcee8183886a5f169b1 | 0.351 | 0.012 |
| e007cfa6ea49f0a7a5009b5def01214b | 0.351 | 0.016 |
| 3481fa43fe5fba6aecdc7f9aae6ed9c0 | 0.328 | 0.031 |
| 5d0c6af420698f1cdff612c8f7960627 | 0.328 | 0.014 |
|  |  |  |
| **(c) Themeda triandra - Bacterial** |  |  |
| **Group Natural #sps. 4** |  |  |
| **ASV** | **IndVal statistic** | **p value** |
| 6110f28a1de867a9983a8a28ecf4e533 | 0.534 | 0.001 |
| dcba105f35d8ebc9e22269c7491ad3a7 | 0.466 | 0.001 |
| 6b94b2c3993ae9c468f7bb2e118dc767 | 0.381 | 0.001 |
|  |  |  |
| **Group Restored #sps. 4** |  |  |
| **ASV** | **IndVal statistic** | **p value** |
| a76e065bdfde0aa883c898ccf5f092fc | 0.469 | 0.001 |
| 9c0de7908dd6ef3d536b42d9e11e0779 | 0.468 | 0.046 |
| e1c7d97fe13e9127d225d76a1feb8c78 | 0.429 | 0.022 |
| 10afda2baef44de4c584a6641de399b1 | 0.396 | 0.043 |

| (d) **Melaleuca quinquenervia - Fungi** |  |  |
| --- | --- | --- |
| **Group Natural #sps. 37** |  |  |
| **ASV** | **IndVal statistic** | **p value** |
| 90e05d3333926ee8560b9c5a503b467c | 0.789 | 0.002 |
| 713ffd93cfa9a81d72a414ae7ba6d9c8 | 0.748 | 0.001 |
| 303e9ab3e4d83037ad04ac4dc6ac3750 | 0.706 | 0.002 |
| dbd67111aa983af089090311480182cf | 0.705 | 0.001 |
| feeabaccf7f7e8bb81fc625d26d59c45 | 0.704 | 0.001 |
| 8e47237dda4929c4b9babc1a1b65c74d | 0.704 | 0.002 |
| 00ced32aa98362b738d91a3eb9bd195a | 0.679 | 0.001 |
| f38a333318715802a5321b77dafa8ac3 | 0.662 | 0.001 |
| 66895503657ee015753be5a5d8f12b97 | 0.662 | 0.001 |
| 195b14b93cac397f8fda702dc4911253 | 0.646 | 0.001 |
| 69963f045eaec775f3c09f76eae92f8b | 0.63 | 0.001 |
| edcde8c88d08002fefa9f17d8ecfea09 | 0.624 | 0.002 |
| c3e71feadae5eaefd11f4d5bc74679db | 0.553 | 0.001 |
| 243047ec9cbadbe0ef97b66bde5de0e0 | 0.537 | 0.001 |
| f3f917fe39b0b06b5bf8e59e6f1f057d | 0.537 | 0.006 |
| ab22bd41b40f836aa59fde3116a5762d | 0.536 | 0.002 |
| 34e8d7ebb543d3d45eeb3b457800e188 | 0.535 | 0.001 |
| 5c77cd1115e7b1db4e9606b1752c70fc | 0.535 | 0.005 |
| fdf8df0e0588c6b6d44bb570afe64370 | 0.533 | 0.028 |
| dd3431bbda24b05a6a3e31d09eba9c20 | 0.526 | 0.003 |
| 531c4bcd01b5e12c1c4068cd346e9ee0 | 0.524 | 0.016 |
| 6d37dfaf83267ed5e21369a52d5afd5d | 0.518 | 0.001 |
| 6fe0ec90b28adf28cfa10f32acb046f5 | 0.499 | 0.003 |
| 1df56c2f60a940dd02d5623b7d7d774d | 0.497 | 0.006 |
| 82e152764a1a9203a9ab95de22170a25 | 0.494 | 0.042 |
| 5b59161ba98bcccdeb8f66e46959f224 | 0.483 | 0.003 |
| bc37a662e480da29bf80a2fcb96659e6 | 0.48 | 0.001 |
| 8b901106999765434f857e5237466ed5 | 0.48 | 0.001 |
| 7ca86a91e6296f55d7eb9c3f24692ac8 | 0.48 | 0.003 |
| c8a0a43d7c33c096b0c30ccf3ce81207 | 0.48 | 0.001 |
| 42cd3aba2a645ef055d779bba22224ce | 0.48 | 0.006 |
| 0fe672eceaca4ab22c9bf7dd5235f06e | 0.479 | 0.002 |
| acd90b1f80b359616afb7d16dbc6fcd9 | 0.474 | 0.005 |
| 5b457d5efc31c2922da5d5e9639606a6 | 0.464 | 0.022 |
| 0f44b5b24fa61635c07fc2fe1259b924 | 0.46 | 0.001 |
| e7f6456d921e809f2d15b343b721d944 | 0.46 | 0.002 |
| e08eecca7c20e8a340ad60b2a4c255c1 | 0.46 | 0.002 |
|  |  |  |
| **Group Restored #sps. 23** |  |  |
| **ASV** | **IndVal statistic** | **p value** |
| 13d714e026ea98d39048d46088bbb27e | 1 | 0.001 |
| 08d6905bddae7391916a2d7dc57bd8ec | 0.998 | 0.001 |
| 1e8aa122bfdb65791884d37c4d488366 | 0.881 | 0.001 |
| 2bb16f07ce02b1b2179b369e196c9175 | 0.809 | 0.001 |
| 4c785ad0f2935d2c52651ecd7a30e6b6 | 0.76 | 0.001 |
| 12a491257ed5f244c249863eaa8131f0 | 0.715 | 0.001 |
| 0a9208a42f7727fb966efea094d574d4 | 0.682 | 0.001 |
| 3fcf8b48adaacfeefa67be0fc7ed748e | 0.65 | 0.001 |
| 204eeef9751b6a75d0ef38d26769aa02 | 0.603 | 0.001 |
| b339d9bf84b94fee469c60751296f76f | 0.597 | 0.001 |
| 8cc20fd55cb59c20fcd556808c6c9d39 | 0.557 | 0.001 |
| 5f58a11393639b4b353ac4843a0d10bf | 0.556 | 0.001 |
| e3059e979256b4d61c50e91573b08a9b | 0.545 | 0.004 |
| e255a7cac19c3236b25e183868983627 | 0.537 | 0.001 |
| 991b9f4a09c941e7927ca29b5036174e | 0.532 | 0.001 |
| da0b6d5a466e9803c1fb91ab10d1db90 | 0.494 | 0.001 |
| 3117663eea7a58dc0ff3303d633869ee | 0.471 | 0.001 |
| ad2fb02dab42cb48fe1a33ea6c6474c0 | 0.463 | 0.015 |
| 936ed71522910d14e4b21ffdedcbcbef | 0.447 | 0.001 |
| 66b09181fa15f98369e57d16b86572e0 | 0.442 | 0.023 |
| 0989f2ca6a943c58dad977967f51adf8 | 0.441 | 0.024 |
| 3a23ecb266f3c4f93eb639fc2de25342 | 0.44 | 0.002 |
| e6304c54bff19aa39f8b5bb48c67e85d | 0.435 | 0.041 |
|  |  |  |
| (e) **Microlaena stipoides - Fungi** |  |  |
| **Group Natural** |  |  |
| **ASV** | **IndVal statistic** | **p value** |
| 524a290c3af922a31db4983ececc3c41 | 0.453 | 0.009 |
| af16991ce12daf846fcbcf604f581dea | 0.424 | 0.029 |
|  |  |  |
| **Group Restored** |  |  |
| **ASV** | **IndVal statistic** | **p value** |
| c834ab12cf6bfcac72f25742be5fadd2 | 0.668 | 0.001 |
| 56b17ee22867f28d69ceed6536bce3f4 | 0.633 | 0.002 |
| ccd4780ec438e2bafad794ca188d894b | 0.607 | 0.001 |
| 78380f34b0cc0fe75299bc0ad51932da | 0.565 | 0.001 |
| cb1f0f2034911892190666c248c43d91 | 0.561 | 0.002 |
| 6830d38a9de8b1b4d97da51e7bf439cd | 0.555 | 0.001 |
| ba28b768ce773adad4cc1a441257b21d | 0.555 | 0.001 |
| 1b77d3c27056146b833598b306411280 | 0.554 | 0.001 |
| ada19fb84a10954399c429afccef6e5e | 0.55 | 0.001 |
| 050e2c7ed6f1586882ad0656a5dd2fe6 | 0.542 | 0.008 |
| 0ebc508ba9b79a722fdabb784c1baabc | 0.529 | 0.002 |
| 858f7224dacd988b893ff394eeea061b | 0.496 | 0.001 |
| 3ec91b1e27aab6fcc891d8102720eb6a | 0.481 | 0.013 |
| 23f38ce51a7a86c4cd7175130a4cf42f | 0.477 | 0.002 |
| 7d7b9bc0ce4c044ef8190f46c7ed0e1b | 0.473 | 0.015 |
| 73f54dd315262f59b0c481e410d19eff | 0.471 | 0.011 |
| 4435b9b859268c6bc83348c6d013a1eb | 0.47 | 0.004 |
| f293dcb354ed13fdb2cd017e835a5b93 | 0.447 | 0.004 |
|  |  |  |
| (f) **Themeda triandra - Fungi** |  |  |
| Group Natural |  |  |
| **ASV** | **IndVal statistic** | **p value** |
| fb85af020c9c974efe524230ec4c13cd | 0.608 | 0.01 |
| 8b0fe00bd6719dfd0b971ce9ea657a06 | 0.534 | 0.001 |
| 7d232f8d9cf50f48c355672f9d1f60c4 | 0.529 | 0.001 |
| 9685d5efab791e0ab92349203828495c | 0.521 | 0.005 |
| ab22bd41b40f836aa59fde3116a5762d | 0.5 | 0.001 |
| c6c303ec40570a32fc80931121416ba7 | 0.5 | 0.001 |
| 997cabda991ca083984f05c60b63350e | 0.486 | 0.02 |
| 5202c68df0068167366946fc5715c851 | 0.482 | 0.001 |
| 6da9d806cdc896a9d344d5922797935a | 0.456 | 0.002 |
|  |  |  |
| **Group Restored** |  |  |
| **ASV** | **IndVal statistic** | **p value** |
| fb7b135d869d1e293ed7087cadf50ab2 | 0.561 | 0.001 |
| fcf701bec0b37d26eaca01060609c805 | 0.533 | 0.002 |
| 050e2c7ed6f1586882ad0656a5dd2fe6 | 0.506 | 0.003 |
| 5d52e596f701c0d7a345f67346ec7d46 | 0.486 | 0.021 |
| 73f54dd315262f59b0c481e410d19eff | 0.43 | 0.049 |
